# Dissociating cognitive and affective empathy across psychopathy dimensions: The role of interoception and alexithymia

**DOI:** 10.3389/fpsyg.2023.1082965

**Published:** 2023-06-29

**Authors:** Carlos Campos, Nuno Barbosa Rocha, Fernando Barbosa

**Affiliations:** ^1^Laboratory of Neuropsychophysiology, Faculty of Psychology and Education Sciences, University of Porto, Porto, Portugal; ^2^Faculty of Medicine, University of Porto, Porto, Portugal; ^3^Neurocognition Group|LabRP, School of Health, Center for Rehabilitation Research, Polytechnic University of Porto, Porto, Portugal; ^4^School of Health, Center for Translational Health and Medical Biotechnology Research, Polytechnic University of Porto, Porto, Portugal

**Keywords:** psychopathy, personality, empathy, social cognition, interoception, interoceptive attention, interoceptive accuracy, alexithymia

## Abstract

This study examined the associations between psychopathy dimensions (triarchic phenotypes and classical factors), empathy domains (cognitive and affective), and interoception (interoceptive attention and accuracy) while accounting for the putative role of alexithymia. A community sample (*n* = 515) completed an online survey encompassing: Triarchic Psychopathy Measure (boldness, meanness, disinhibition); Levenson Self-Report Psychopathy Scale (primary and secondary psychopathy); Body Perception Questionnaire (interoceptive attention); Interoceptive Accuracy Scale; Toronto Alexithymia Scale. Hierarchical linear regression models were implemented for hypothesis-driven analyses examining the associations between psychopathy, empathy, and interoception while controlling for sex, age, and alexithymia. Exploratory path models were employed to investigate alexithymia and/or cognitive empathy as mediators between interoception and psychopathy. Our results largely confirmed the postulated empathy profiles across psychopathy dimensions, as meanness and primary psychopathy displayed a broad empathy impairment, while disinhibition and secondary psychopathy were only associated with diminished cognitive empathy. Importantly, boldness displayed a unique pattern (enhanced cognitive empathy and reduced affective empathy), further reinforcing its importance within the constellation of psychopathy traits. Contrary to our hypotheses, self-perceived interoceptive attention and accuracy were not associated with either psychopathy dimension after controlling for alexithymia. However, interoceptive accuracy and alexithymia were associated with cognitive empathy, while alexithymia was also positively related to all psychopathy dimensions (as expected), despite the unexpected strong and negative association with boldness. Exploratory analyses suggested significant indirect effects (mediation) between interoceptive accuracy and psychopathy *via* alexithymia and/or cognitive empathy. These mediating effects must be interpreted with caution and future studies should be designed to formally test this model.

## 1. Introduction

Psychopathy is a multidimensional personality structure encompassing a wide constellation of traits that complexly interact with each other and with several psychologically meaningful constructs (Skeem et al., 2011; Lilienfeld, 2018; Sellbom and Drislane, 2021). This view is vastly consensual nowadays, as psychopathy traits are considered to be continuously distributed across the population, with each subject displaying a unique multidimensional profile of features. The specific subdimensions underlying psychopathy are far from being consensual, although two major conceptual models are currently discussed. The classical 2-factor model (Hare et al., 1990; Levenson et al., 1995; Hare and Neumann, 2008) considers that psychopathy encompasses two independent (yet correlated) dimensions, namely interpersonal-affective traits (*Factor 1/primary psychopathy*—superficial charm, manipulation, callousness, absence of remorse, shallow affect) and impulsive-antisocial features (*Factor 2/secondary psychopathy*—impulsivity, lack of behavioral control, criminal versatility, recidivism). Criminal behavior presents a central role within this model, as psychopathy traits are considered to be intrinsically linked to antisocial manifestations from a genetic and longitudinal standpoint (Hare and Neumann, 2008). Despite the important contribution of this model to psychopathy research, the centrality of criminal behavior has been somewhat questioned. The strong focus on behavioral traits can arguably make the psychopathy construct overinclusive and non-specific, as impulsive and antisocial tendencies also co-occur with several externalizing disorders (Krueger et al., 2002, 2007; Nelson and Foell, 2018). Furthermore, these factors were originally proposed within criminal samples, likely leading to an over-inclusion of maladaptive traits vs. under-inclusion of adaptive expressions of psychopathy (Cooke and Michie, 2001; Skeem et al., 2011). These adaptive manifestations, described since the early descriptions of psychopathy (Cleckley, 1941, 1976), are presumably observed in the now-named “successful psychopath,” defined as an individual who displays high levels of psychopathy but is still capable of sustaining normal daily functioning (Gao and Raine, 2010; Steinert et al., 2017; Gao et al., 2020; Bronchain et al., 2021).

To address these caveats, the triarchic model emerged as an alternative multidimensional framework that proposed the exclusion of antisociality and the inclusion of adaptive traits within the psychopathy personality structure (Patrick et al., 2009; Patrick and Drislane, 2015). The triarchic model proposes three dissociable (despite interrelated) phenotypic dispositions, namely *boldness* (fearless-dominant proclivities - low-stress reactivity, social dominance, persuasiveness, venturesomeness), *meanness* (callous-aggression tendencies—deficient empathy, lack of affiliative capacity, predatory exploitativeness, empowerment through cruelty), and *disinhibition* (externalizing proneness—impulsivity, impaired emotional regulation, lack of planning, hostility). Within this model, antisocial behavior is not included as a core feature of psychopathy, although meanness and disinhibition capture features considered proximal to antisociality. Hence, psychopathy is viewed within a hierarchical framework, where interpersonal-affective traits may (or may not) constitute a risk factor for antisocial behavior contingent on the influence of other individual and contextual factors (Cooke and Michie, 2001; Cooke et al., 2006; Skeem et al., 2007; Patrick et al., 2009; Skeem and Cooke, 2010). Despite the contribution of the triarchic model to foster research on the etiological and neurobiological pathways underlying psychopathy dimensions (Patrick et al., 2012), it has also faced some criticism regarding the importance of boldness-related traits within the construct (Miller and Lynam, 2012; Marcus et al., 2013) and the reliability of its 3-factor structure (Roy et al., 2021).

The role of antisocial behavior within psychopathic personality is still a matter of discussion and division nowadays. Importantly, the classical 2-factors and the triarchic phenotypes provide distinct operationalizations of the interpersonal, affective, and behavioral manifestations of psychopathy. This divergence should be accounted for when producing evidence examining how psychopathy dimensions interact with other psychological constructs.

### 1.1. An overview of empathy within psychopathy traits

Empathy, historically a hallmark of psychopathy, is also widely considered a multidimensional construct across most conceptualizations (Eklund and Meranius, 2021). Despite the ongoing debate regarding its specific subdomains and underlying processes, nowadays it is safe to argue that most models (e.g., Reniers et al., 2011; Zaki and Ochsner, 2012; Dvash and Shamay-Tsoory, 2014; de Waal and Preston, 2017) converge on the fact that empathy encompasses at least two major interconnected domains: *cognitive empathy*, defined as the ability to infer the mental states (with or without affective content); *affective empathy*, conceptualized as the capacity of being sensitive to and vicariously experiencing the emotional states felt by others. Recent functional neuroimaging meta-analyses have provided further robust evidence for the existence of two brain networks that separately (although interactively) support cognitive or affective empathic processing (Kogler et al., 2020; Schurz et al., 2021).

The putative differential role of cognitive and affective processes on social behavior has been discussed since the early descriptions of psychopathy. Cleckley (1941, 1976) described the so-called emotion paradox, suggesting that highly psychopathic individuals were able to effectively understand emotional information despite their inability to use this information to guide their behavior. This dual-process route for processing social–emotional information has been largely confirmed by meta-analytical evidence indicating intact explicit cognitive processing of other’s distress in psychopathy, despite the co-occurring impairment in affective responsivity at the automatic-visceral level (Hoppenbrouwers et al., 2016). Similarly, other authors have proposed that reduced affective empathy is a core feature observed across all psychopathy dimensions, although interpersonal traits might be associated with intact or even enhanced cognitive empathy (Gao and Raine, 2010; Gao et al., 2020).

The broad empathy impairment within the callous-affective traits of psychopathy has been meta-analytical confirmed (Northam and Dadds, 2020; Waller et al., 2020), but until recently there was no comprehensive evidence examining the interplay between all psychopathy dimensions and empathy domains. Burghart and Mier (2022) reported scale-specific analyses assessing how specific empathy subprocesses (e.g., empathic concern, perspective-taking, personal distress) interact with several psychopathy questionnaires, reporting a broad empathy impairment in primary and secondary psychopathy, contrasting with complex empathy profiles across triarchic-based questionnaires. Meta-analytical evidence from Campos et al. (2022) indicated that classical 2-factors were negatively related to both empathy domains (effect sizes larger for primary psychopathy), while triarchic phenotypes presented three distinct empathy profiles. Meanness was associated with a clear and broad empathy impairment, while disinhibition displayed smaller effect sizes for both empathy domains. Contrastingly, boldness was unrelated to cognitive empathy, despite the negative association with the affective domain. These recent meta-analyses provided extremely important contributions, despite still having the limitation of not being able to evaluate how empathy domains interact with different psychopathy conceptualizations within the same sample. Moreover, available evidence still relies largely on the Interpersonal Reactivity Index (Burghart and Mier, 2022; Campos et al., 2022), an empathy questionnaire that has been somewhat questioned lately regarding its factor structure, construct validity of its specific subscales (e.g., Personal Distress), and its suitability to adequately operationalize cognitive and affective empathy (Chrysikou and Thompson, 2016; Murphy B. A. et al., 2020; Wang et al., 2020).

Altogether, existing conceptual and meta-analytical evidence suggests a broad empathy impairment within the affective dimension of psychopathy, while empathy does not seem to be reliably associated with behavioral traits (secondary psychopathy and disinhibition). Conversely, enhanced cognitive empathy within interpersonal traits (e.g., social dominance, manipulate behavior) may allow to compensate for affective empathy deficits within this dimension. Importantly, the triarchic model clearly dissociates interpersonal and affective traits (boldness and meanness phenotypes, respectively), while these dimensions are clustered together within primary psychopathy in the classical 2-factor model. Ultimately, there is still a demand for studies assessing how competing conceptualizations of psychopathy dimensions interact with empathy measures that effectively dissociate cognitive and affective empathy.

### 1.2. Interoception: The missing link between psychopathy and empathy?

Recent proposals addressing the underlying mechanisms of social cognition could also provide additional insights regarding the complex interplay between psychopathy and empathy. Interoception, broadly defined as the perception of internal bodily stimuli, may be a putative candidate to further explore this. The boundaries and conceptual frameworks for interoception measurement are still not universally accepted, but recently Murphy et al. (2019) proposed a 2 × 2 factorial framework that provides a solid and testable tool for measuring individual differences in interoception. Within this model, the first factor targets which construct is being measured, namely *interoceptive accuracy* (the ability to accurately perceive interoceptive signals) and *interoceptive attention* (the degree to which interoceptive signals are the object of attention). The second factor addresses how the previously described constructs are being measured, that is, self-reported beliefs vs. objective performance measures. Importantly, there has been recent evidence from several countries highlighting specific self-report questionnaires that can index beliefs regarding either interoceptive attention or accuracy (Murphy J. et al., 2020; Campos et al., 2021; Brand et al., 2022; Gabriele et al., 2022; Tünte et al., 2022).

The link between interoception and empathy has been driven by compelling meta-analytical results indicating the convergence of the neural correlates associated with interoception, emotion, and social information processing (Adolfi et al., 2017). Behavioral studies have been somewhat inconsistent, despite increasingly suggesting that interoceptive accuracy is associated with cognitive empathy (Handford et al., 2013; Ainley et al., 2014; Tajadura-Jiménez and Tsakiris, 2014; Grynberg and Pollatos, 2015; Shah et al., 2017; Shaw et al., 2020; Baiano et al., 2021). Contrastingly, evidence addressing the role of interoceptive attention within empathic processing is much scarcer and more limited (Morganti et al., 2020). Regardless, it is growingly accepted that interoception may be connected not only to self-related affective processing but also to our ability to perceive and share the emotional states of others (Ainley et al., 2016; Barrett, 2016; Ferreira-Santos, 2016; Seth and Friston, 2016; Fotopoulou and Tsakiris, 2017; Ondobaka et al., 2017; Marshall et al., 2018).

With this in mind, it is feasible to postulate that interoception variations within specific psychopathy dimensions may modulate the ability to perceive somatic sensations that signal emotional valence within social interactions (Hoppenbrouwers et al., 2016; Gao et al., 2019). Importantly, several neurobiological models have implicated key interoception-related brain regions within the core network underlying the emotional processing deficits associated with psychopathy (Fowles and Dindo, 2009; Patrick et al., 2012; Blair, 2013). So far, there have been only a handful of studies exploring the link between psychopathy and interoception (Nentjes et al., 2013; Zwets et al., 2014; Lyons and Hughes, 2015; Lamoureux and Glenn, 2021), and the existing evidence presents several limitations. First, most of these only provided findings regarding offenders, which is not ideal for fully capturing the dimensional nature of psychopathy. Secondly, none of the studies included the triarchic model, consequently neglecting the more adaptive expressions of psychopathy. Thirdly, none of the authors applied a formal theoretical framework to measure individual differences in interoception such as the previously described 2 × 2 factorial model.

### 1.3. The putative confounding role of alexithymia, age, and sex

When discussing the psychopathy-empathy-interoception tripartite interaction, it is important to consider the putative role of additional confounding variables. Within this scope, alexithymia emerges as an important subclinical construct that should be accounted for. Alexithymia can be broadly described as difficulties to identify, describe, and interpret one’s own emotional experiences combined with externally-oriented thinking (Bagby et al., 1994a,b). Importantly, alexithymia has been systematically linked to the core constructs of the current work. A recent meta-analysis indicated that alexithymia is positively associated with total psychopathy scores as well as with the classical interpersonal-affective and impulsive-antisocial factors of psychopathy (Burghart and Mier, 2022). Pisani et al. (2021) conducted a comprehensive systematic review that suggests that alexithymia is related to an impaired ability to infer the emotional states of others. Finally, there is also meta-analytical evidence suggesting that self-reported interoceptive attention is positively associated with alexithymia, while the opposite pattern is observed when considering self-reported interoceptive accuracy (Trevisan et al., 2019). The robust evidence highlighting the role of alexithymia within psychopathy, empathy, and interoception strongly suggests that this variable should be accounted for when examining the interaction between these three constructs.

Additionally, sex- and age-related effects should also be accounted for when considering the psychopathy, empathy, and interoception interplay. The enhanced prominence of psychopathy in males vs. females has been meta-analytically validated, even after controlling for other personality traits (Muris et al., 2017; Sanz-García et al., 2021). Sex-empathy interaction is far more intricate, as current evidence suggests that sex differences may (or may not) arise from a complex interaction between methodological (self-report vs. experimental tasks), biological, and environmental factors (Konrath et al., 2011; Christov-Moore et al., 2014; Warrier et al., 2018; Abramson et al., 2020; Rochat, 2022; Zhao et al., 2022). Recent meta-analytical evidence also indicated that male subjects display enhanced cardiac interoceptive accuracy, despite findings being consistent with other interoceptive modalities (Prentice and Murphy, 2022). Finally, there is also comprehensive evidence suggesting small (but significant) larger alexithymia scores in male subjects (Levant et al., 2009). Regarding the putative confounding role of age, several authors have reported a decrease in psychopathy traits across the life span, although interpersonal-affective traits remain more or less stable across time within offender samples (Harpur and Hare, 1994; Huchzermeier et al., 2008; Gill and Crino, 2012; Maurer et al., 2022). Similarly, there is also evidence indicating that empathy domains (cognitive vs. affective) seemingly display different development patterns across the lifespan (Sun et al., 2018; Beadle and de la Vega, 2019; Main and Kho, 2020). Ultimately, age-related decline in both self-perceived and objectively measured interoceptive abilities has also been suggested by several authors, despite findings seemingly being contingent on the selected assessment procedures (Khalsa et al., 2009; Murphy et al., 2018; Nusser et al., 2020; MacCormack et al., 2021). Altogether, these findings provide robust evidence for the need to account for sex and age within the context of the current work.

### 1.4. Study goals and hypothesis

Considering the previously described gaps in the field, the overarching goal of this study is to investigate the complex interplay between psychopathy, empathy, and interoception. More specifically, the current work aims to examine how psychopathy dimensions, as conceptualized by two major theoretical frameworks (triarchic model—boldness, meanness, and disinhibition vs. classical factors—primary and secondary psychopathy), are differentially associated with specific empathy domains (cognitive and affective) and interoception measures (self-reported interoceptive attention and accuracy). Furthermore, we will also investigate how cognitive and affective empathy are related to interoceptive attention and accuracy. Importantly, considering the previously described putative confounders, the current study will also examine whether the associations between psychopathy, empathy, and interoception are retained after controlling for sex, age, and alexithymia.

Below, we provide an overview of the preregistered hypotheses for the current study. According to the previously described conceptual and meta-analytical evidence, we postulated three distinct empathy profiles for the triarchic phenotypes of psychopathy. Meanness ought to be associated with a broad empathy impairment, in contrast with boldness and disinhibition which should display specific negative associations with affective and cognitive empathy, respectively. Importantly, cognitive empathy should be enhanced in the boldness phenotype, with disinhibition being positively associated with the affective domain. Contrastingly, the fine-grained empathy domain dissociation was not hypothesized for the classical 2-factors, as both cognitive and affective empathy should be impaired in primary and secondary psychopathy.

Differential associations were also postulated between empathy domains and interoception measures. As previously described, existing evidence suggests that interoceptive accuracy is positively related to cognitive empathy. Despite the scarce evidence regarding interoceptive attention, we argue for its positive association with affective empathy, as the enhanced allocation of attentional resources to interoceptive stimuli should also increase the proneness to detect the bodily signals triggered by the observing the emotional experiences of others, thus maximizing the likelihood of affective sharing.

The specific interaction between empathy domains and interoception measures consequently guided our hypotheses regarding the role of interoception across psychopathy dimensions. As interoceptive accuracy has been linked to enhanced cognitive empathy, it should also be positively related to boldness, while being negatively associated with meanness, disinhibition, and both classical psychopathy factors. Conversely, interoceptive attention (postulated to be linked to affective empathy) should thus be enhanced in disinhibition, while being impaired across boldness, meanness, primary and secondary psychopathy.

Although univariate analyses and formal correlation comparisons will be first conducted to examine the previously described hypotheses, further analyses accounting for putative confounders will also be implemented. Firstly, partial correlations will be used to control for alexithymia, as we postulated that this construct should be negatively associated with cognitive empathy and interoceptive accuracy while being positively related with interoceptive attention and several psychopathy dimensions (meanness, disinhibition, primary and secondary psychopathy). Secondly, as several sex differences (females with enhanced empathy and interoceptive attention; males with higher interoceptive accuracy, alexithymia. and psychopathy traits—except for disinhibition) and age-related effects (negative association with cognitive empathy, interoception measures, primary and secondary psychopathy) were expected, multivariate models were implemented to account for these variables, as well as for alexithymia and the covariance between the predictors. We postulated that the previously described associations between psychopathy dimensions, empathy domains, and interoception measures should be retained after controlling for sex, age, and alexithymia. Lastly, as this was the first study to formally examine how specific measures targeting self-reported interoceptive attention and accuracy interact with psychopathy and empathy, exploratory mediation analyses (contingent on results from confirmatory testing) were also conducted to check for possible mediation effects between the previously described constructs.

## 2. Method

The current study was preregistered and full methodological details can be found at https://osf.io/5jhcw. Datasets and full outputs from the analyses are available at https://osf.io/zyf4e/.

### 2.1. Participants

A community sample of 515 subjects aged between 18 and 72 (*M*_age_ = 30.74; *SD* = 10.52; 59.61% female) was recruited (without any compensation for participation) using a non-list based, non-probability sample (social media advertising and personal contacts for snowball sampling) to maximize the sociodemographic heterogeneity of participants (Callegaro et al., 2015). Within this approach, sample recruitment is spread as broadly as possible, using several recruitment channels to target different communities. Summary statistics for sociodemographic data were accessed periodically to screen whether recruited participants were heterogeneous regarding several variables (e.g., sex, age, educational level). This allowed for subsequent advertising strategies to target specific communities of participants (e.g., in-person contacts to reach older subjects who are less represented on social media). Table 1 presents the characteristics of the participants included in the current study.

**Table 1:** Sample characteristics (including descriptive statistics of questionnaires scores).

	M (SD)	Min–Max
Age	30.74 (10.52)	18–72
Education (years)	15.03 (2.99)	1–25
TriPM Boldness	28.18 (8.13)	2–49
TriPM Meanness	9.47 (6.6)	0–35
TriPM Disinhibition	15.25 (7.01)	1–40
TriPM Total	52.9 (14.68)	15–105
LSRP Primary	27.02 (5.92)	16–46
LSRP Secondary	20.74 (4.43)	11–36
LSRP Total	47.76 (8.33)	30–77
QCAE Cognitive	58.98 (7.37)	35–76
QCAE Affective	33.1 (5.11)	17–44
QCAE Total	92.08 (10.09)	64–120
BPQ Body Awareness	78.02 (22.38)	33–130
IAS	85.01 (10.92)	55–105
TAS	50.2 (11.22)	25–82
	***n* (%)**	
**Sex**		
Females	307 (59.6%)	
Males	208 (40.4%)	
**Education (categorical)** ^*^		
Elementary School	2 (0.4%)	
Middle School	21 (4.1%)	
High School	129 (25.0%)	
Undergraduate Degree	243 (47.2%)	
Master’s Degree	116 (22.5%)	
Doctoral Degree	4 (0.8%)	
Psychiatric history	89 (17.3%)	
Neurological history	12 (2.3%)	
Non-native speakers	6 (1.2%)	

* 158 subjects (30.7%) were college students at the time of survey completion.

### 2.2. Instruments

#### 2.2.1. Triarchic Psychopathy Measure

The Triarchic psychopathy measure Triarchic Psychopathy Measure (TriPM) was developed to assess psychopathy dimensions according to the triarchic model (Patrick, 2010), including 19 items for *boldness*, 19 for *meanness*, and 20 for *disinhibition*. Each item is scored on a 4-point Likert scale (True, Somewhat True, Somewhat False, False) with larger scores indexing higher psychopathy traits. This scale was adapted and psychometrically tested for Portuguese (Vieira et al., 2014; Paiva et al., 2020). In the current sample, each phenotype subscale displayed good internal consistency (boldness ω_categorical_ = 0.852; meanness ω_categorical_ = 0.879; disinhibition ω_categorical_ = 0.829), with an excellent value observed for the total score (ω_categorical_ = 0.922).

#### 2.2.2. Levenson Self-Report Psychopathy Scale

Developed by Levenson et al. (1995) and adapted to Portuguese by Barbosa et al. (2014), the Levenson Self-Report Psychopathy Scale (LSRP) was developed to assess the 2-classical factors of psychopathy in non-forensic samples (16 items for *primary psychopathy*; 10 items for *secondary psychopathy*). Each item is rated on a 4-point Likert scale ranging from strongly disagree (1) to strongly agree (4), with larger scores representing higher psychopathy traits. Primary (ω_categorical_ = 0.821) and total psychopathy (ω_categorical_ = 0.839) presented good reliability, while secondary psychopathy scores were somewhat questionable within our sample (ω_categorical_ = 0.692).

#### 2.2.3. Questionnaire of Cognitive and Affective Empathy

Self-report measure that considers the multidimensional nature of empathy, namely the cognitive and affective empathy domains (Reniers et al., 2011). The Questionnaire of Cognitive and Affective Empathy (QCAE) was selected as it provides the most suitable alternative to index cognitive and affective empathy accordingly to more contemporary frameworks. From a conceptual standpoint, the QCAE was developed to clearly dissociate the ability to infer the emotional states of others (cognitive empathy) vs. being sensitive to or vicariously experiencing those feelings (affective empathy). Moreover, neuroimaging evidence suggests that QCAE scores are differentially associated with the core brain networks typically associated with cognitive and affective empathy (Eres et al., 2015). The European Portuguese version of the QCAE (Queirós et al., 2018) includes 30 items answered on a 4-point Likert scale (strongly agree, slightly agree, slightly disagree, and strongly disagree), with higher scores indicating greater empathy. The questionnaire includes 19 items for *cognitive empathy*, which can be further subdivided into *perspective-taking* (10 items) and *online simulation* (9 items), and 11 items for *affective empathy*, which encompasses the *emotion contagion* (4 items), *proximal responsivity* (4 items), and *peripheral responsivity* (3 items) subscales. In our sample, the internal consistency was excellent for cognitive (ω_categorical_ = 0.956), affective (ω_categorical_ = 0.908), and total empathy scores (ω_categorical_ = 0.991). Concerning the 5 subscales, both perspective-taking (ω_categorical_ = 0.874) and online simulation (ω_categorical_ = 0.850) presented good internal consistency, with adequate values observed for emotion contagion (ω_categorical_ = 0.734) and peripheral responsivity (ω_categorical_ = 0.791), while proximal responsivity was questionable (ω_categorical_ = 0.658). Regardless, only cognitive and affective empathy scores were used for confirmatory analyses in the current work (see Supplementary material 1 for zero-order correlations pertaining to 5-factor subscales).

#### 2.2.4. Body Perception Questionnaire - Body Awareness

Developed by Cabrera et al. (2018), this questionnaire includes 26 items that can be used to measure interoceptive attention, as suggested by Murphy et al. (2019), with higher scores indicating that subjects consider that interoceptive inputs are often the target of their attention. The Body Perception Questionnaire (BPQ) includes a second domain (BPQ Autonomic Reactivity) that was not used for the purpose of the current study (see Supplementary material 1 for exploratory analyses). It is also important to highlight that, although Cabrera et al. (2018) used the binary scoring system (0 or 1 for each item) for the psychometric analysis of the BPQ, scoring for the current study was completed using the full-item responses (5-point Likert scale ranging from Never to Always), which was originally recommended to allow larger sensitivity for individual differences (Porges et al., 1993/2015). The data collected here was also used to validate the Portuguese version of the BPQ which also provided further evidence for using full-item response instead of binary scoring (Campos et al., 2021). Within the current sample, BPQ Body Awareness and BPP Autonomic Reactivity both displayed excellent internal consistency (ω_categorical_ = 0.978 and 0.944, respectively).

#### 2.2.5. Interoceptive Accuracy Scale

This scale includes 21 items (5-point Likert scale, ranging from strongly agree to strongly disagree) to assess self-reported interoceptive accuracy, with higher scores implying enhanced accuracy in perceiving interoceptive signals (Murphy J. et al., 2020). The Interoceptive Accuracy Scale (IAS) was also validated in European Portuguese using data stemming from the current study (Campos et al., 2021), displaying excellent internal consistency (ω_categorical_ = 0.970).

#### 2.2.6. Toronto Alexithymia Scale

Self-report scale assessing alexithymia, comprising 20 items rated on a 5-point Likert scale ranging from strongly agree (5) to strongly disagree (1). Within the current work, only the total alexithymia score was used for confirmatory analyses (higher scores indicate greater alexithymia traits), despite this scale encompassing three subscales, namely difficulty *identifying feelings* (7 items), *difficulty describing feelings* (5 items), and *externally-oriented thinking* (8 items). This questionnaire was originally developed by Bagby et al. (1994a,b) and adapted to Portuguese by Prazeres et al. (2000). In the current study, Toronto Alexithymia Scale (TAS) total score displayed good internal consistency (ω_categorical_ = 0.847). Within the three subscales, only identifying feelings and difficulty describing feelings presented at least acceptable consistency (ω_categorical_ = 0.847 and 0.797, respectively), while externally-oriented thinking displayed a blatantly unacceptable score (ω_categorical_ = 0.493). Within the scope of our hypothesis-driven analyses, only total alexithymia scores were used, although exploratory correlational analyses with subscale scores are reported in Supplementary material 1.

### 2.3. Procedures

This study was approved by the institutional Ethics Committee and Data Protection Officer. The data was collected online through LimeSurvey v3.22.18 + 200,603. Participants read an online briefing about the study and completed an electronic consent form before starting the survey. Instruments were presented in a randomized order (after the consent form and sociodemographic questions) to prevent order effects.

### 2.4. Statistical analysis

The previously reported categorical omega coefficients (ω_categorical_), more suitable for ordered-categorical items (Kelley and Pornprasertmanit, 2016), were computed as measures of internal consistency for questionnaire scores. Internal consistency was classified as suggested by Kline (2016): < 0.50 unacceptable; ≥ 0.50 and < 0.60 poor; ≥ 0.60 and < 0.70 questionable; ≥ 0.70 and < 0.80 adequate/acceptable; ≥ 0.80 and < 0.90 good; ≥ 0.90 excellent. Regarding assumption testing, the normality assumption was formally defined using threshold criteria for skewness and kurtosis—less than |2.0| and |9.0|, respectively (Gignac, 2019). Homogeneity of variance was tested using Levene’s F test (parametric or non-parametric version, contingent on data distribution—Nordstokke and Zumbo, 2010; Nordstokke et al., 2011). For regression models, the following assumptions were examined, as recommended by Gignac (2019): linearity (visualization of residual plots), normally distributed residuals (previously described skewness and kurtosis criteria), influential cases (Cook’s distance > 1), homoscedasticity (Koenker test; Daryanto, 2020), collinearity (*r* > 0.95 considered problematic), and multicollinearity (variance inflation factor > 10).

Confirmatory statistical testing included independent samples *t*-tests (Welch tests for heterogeneity of variances), zero-order and partial correlations, and hierarchical linear regression models. Independent-samples t-tests (or Welch tests) were used for sex comparison, with effect sizes computed using Hedges’ *g* (Glass’ Δ for Welch test) and classified as suggested by Cohen (1988): small = |0.20|, medium = |0.50| and large = |0.80|. Zero-order correlation coefficients were utilized to examine the associations between psychopathy, empathy, interoception, alexithymia, and age. Hypothesized differences between the correlations were formally tested using Steiger’s Z-test for dependent correlations (Steiger, 1980) *via* the quantpsy web implementation (Lee and Preacher, 2013). Partial correlations were used to check whether the associations between psychopathy, empathy, and interoception were retained after controlling for alexithymia. Correlation coefficients were classified as small, *r* ≥ |0.10|, medium, *r* ≥ |0.20|, and large, *r* ≥ |0.30|, as suggested by Gignac and Szodorai (2016).

Hierarchical linear regression models (10 models) were used to test several hypotheses regarding the interplay between psychopathy, empathy, and interoception. Importantly, the putative confounding role of sociodemographics (sex and age) and alexithymia was accounted for by always including these variables in the first and second block of each model, respectively. Firstly, specific models were implemented to examine the association of psychopathy dimensions (triarchic phenotypes or classical factors—included in the last block of each model) with either cognitive empathy (Models 1 and 2) or affective empathy (Models 3 and 4). Within these models, psychopathy dimensions were included as predictors to account for the covariance between triarchic phenotypes (Models 1 and 3) or between primary and secondary psychopathy (Models 2 and 4). Secondly, Models 5 and 6 were employed to specifically evaluate how interoception measures (interoceptive attention and accuracy—inserted in the final block of each model) predicted either cognitive or affective empathy, respectively. The last set of models examined how psychopathy traits (triarchic phenotypes or classical factors—included in the last block of each model to account for their covariance) predict either interoceptive attention (Models 7 and 8) or interoceptive accuracy (Models 9 and 10). Across these models, wild bootstrapping was used to estimate *p-*values if the assumption of homoscedasticity was not met.

In addition to confirmatory testing, exploratory analyses were implemented using path models to examine the interplay between all variables of interest. The specific direct and indirect effects were proposed by combining the preregistered hypotheses with existing theoretical knowledge and the results stemming from confirmatory analyses (detailed rationale in the Results section). Path models were estimated using maximum likelihood estimation and bootstrapping with 5,000 resamples. Effects were thus computed with 99% bias-corrected confidence intervals (significance threshold 0.01). Univariate normality was assessed using the previously described skewness and kurtosis threshold, while multivariate normality was evaluated using multivariate kurtosis (values > 5 indicative of departure from normality). The following indicators were used to describe the absolute model fit: Chi-squared goodness-of-fit statistic (significance); Comparative Fit Index (CFI; ≥ 0.90 acceptable fit; ≥ 0.95 good fit); Tucker Lewis Index (TLI; ≥ 0.90 acceptable fit; ≥ 0.95 good fit); Root Mean Square Error of Approximation (RMSEA; ≤ 0.08 acceptable fit; ≤ 0.06 with 90% CIs ≤ 0.10 good fit). Relative model fit (model comparison) was assessed using the Akaike Information Criterion (AIC) and Bayesian Information Criterion (BIC)—lower values indicating better fit. Further exploratory endeavors included correlation analyses using the QCAE and TAS subscale scores as well as the BPQ Autonomic Reactivity—see Supplementary material 1.

Statistical analyses were implemented using SPSS Statistics and AMOS v28 with alpha set at 0.01. Categorical omega coefficients and the confidence intervals for partial correlations were computed using R packages (*MBESS* and *bigstatsr*, respectively). All the previously described statistical procedures were replicated (control analyses) excluding: careless respondents (*n* = 8), identified using a response time cut-off criterion (less than 3 s per item on at least two of the completed questionnaires) to exclude unrealistically fast respondents that produce poor data quality (Huang et al., 2012; Maniaci and Rogge, 2014; Niessen et al., 2016); univariate outliers (*n* = 1), screened using the 3 interquartile range criteria due to the large sample size; multivariate outliers (*n* = 1), detected using Mahalanobis distance and only observed in the path models; possible confounders (non-native speakers, psychiatric and/or neurologic disorders; *n* = 99), which were mainly due to subjects with self-reported psychiatric disorders (*n* = 89).

## 3. Results

### 3.1. Sex comparison and correlational analyses

Full univariate results can be found in Tables 2, 3. Additionally, formal comparisons between correlations and partial correlations can be found in Tables 4, 5, respectively. Regarding sex comparison, male subjects displayed larger psychopathy traits on all total and subscale scores (all *p* < 0.001, *g* = [0.394, 0.748], Δ = [0.328, 0.943]),^1^ except on LSRP Secondary, *t* = −0.043, *p* = 0.966, *g* = 0.004. Female participants displayed higher scores on QCAE Affective, *t* = 7.216, *p* < 0.001, *g* = 0.647, QCAE Total, *t* = 4.893, *p* < 0.001, *g* = 0.439, and BPQ Body Awareness, *t* = 3.173, *p* = 0.002, *g* = 0.285. Considering age-related effects, negative correlations were found with LSRP Primary and total scores, QCAE Affective, and BPQ Body Awareness, although effect sizes were small (all *p* ≤ 0.008, *r* = [−0.157, −0.117]). Hence, although the previously described sex- and age-related hypotheses were only partially supported, evidence from univariate analyses indicates that these sociodemographic variables are associated with psychopathy, empathy, and/or interoception, as should thus be accounted for in multivariate models.

**Table 2:** Sex comparison—independent samples *t*-tests (Welch’s tests for heterogeneity of variances).

	Age	TriPM Boldness	TriPM Meanness	TriPM Disinhibition	TriPM Total	LSRP Primary	LSRP Secondary	LSRP Total
Female (*n* = 307) *Mean (SD)*	29.00 (9.98)	25.87 (7.68)	7.40 (5.32)	14.22 (6.25)	47.50 (12.36)	25.72 (5.57)	20.73 (4.47)	46.46 (8.12)
Male (*n* = 208) *Mean (SD)*	33.30 (10.79)	31.59 (7.56)	12.53 (7.12)	16.77 (7.77)	60.89 (14.21)	28.94 (5.91)	20.75 (4.39)	49.69 (8.29)
Mean Diff. *99% CIs*	−4.30 [−6.69, −1.90]	−5.72 [−7.49, −3.95]	−5.13 [−6.55, −3.71]	−2.55 [−4.15, −0.95]	−13.39 [−16.45, −10.34]	−3.21 [−4.54, −1.89]	−0.02 [−1.05, 1.01]	−3.23 [−5.13, −1.33]
*t*	−4.640	−8.344	−8.852	−3.945	−11.054	−6.273	−0.043	−4.394
*p*	< 0.001	< 0.001	< 0.001	< 0.001	< 0.001	< 0.001	0.966	< 0.001
Hedges g *Glass’ Δ	0.416	0.748	0.721*	0.328*	0.943*	0.562	0.004	0.394
	QCAE Cognitive	QCAE Affective		QCAE Total	BPQ Body Awareness	IAS		TAS
Female (*n* = 307) *Mean (SD)*	59.46 (7.11)	34.37 (4.74)		93.83 (9.61)	80.57 (21.36)	84.49 (11.25)		50.62 (11.35)
Male (*n* = 208) *Mean (SD)*	58.27 (7.70)	31.22 (5.05)		89.49 (10.25)	74.25 (23.35)	85.77 (10.39)		49.6 (11.01)
Mean Diff. *99% CIs*	1.18 [−0.53, 2.89]	3.16 [2.03, 4.29]		4.34 [2.05, 6.63]	6.32 [1.17, 11.47]	−1.29 [−3.82, 1.25]		1.02 [−1.58, 3.62]
*t*	1.790	7.216		4.893	3.173	−1.312		1.012
*p*	0.074	< 0.001		< 0.001	0.002	0.190		0.312
Hedges g *Glass’ Δ	0.160	0.647		0.439	0.285	0.118		0.091

**Table 3:** Zero-order correlations.

	Age (years)	TriPM Boldness	TriPM Meanness	TriPM Disinhibition	TriPM Total	LSRP Primary	LSRP Secondary	LSRP Total	QCAE Cognitive	QCAE Affective	QCAE Total	BPQ Body Awareness	IAS	TAS
Age (years)	1	0.089 [−0.025, 0.200]	−0.037 [−0.150, 0.077]	0.010 [−0.103, 0.123]	0.038 [−0.076, 0.150]	−0.125 [−0.235, −0.012]	−0.053 [−0.165, 0.061]	−0.117 [−0.227, −0.003]	−0.005 [−0.118, 0.108]	−0.117 [−0.228, −0.004]	−0.063 [−0.175, 0.051]	−0.157 [−0.266, −0.045]	0.081 [−0.033, 0.192]	−0.097 [−0.208, 0.017]
TriPM Boldness	0.043	1	0.234 [0.124, 0.338]	−0.142 [−0.252, −0.029]	0.591 [0.511, 0.660]	0.204 [0.093, 0.310]	−0.250 [−0.353, −0.141]	0.012 [−0.101, 0.125]	0.190 [0.079, 0.297]	−0.269 [−0.371, −0.161]	0.003 [−0.111, 0.116]	−0.070 [−0.182, 0.044]	0.133 [0.020, 0.243]	−0.414 [−0.504, −0.316]
TriPM Meanness	0.404	<0.001	1	0.519 [0.431, 0.597]	0.827 [0.787, 0.859]	0.628 [0.554, 0.692]	0.374 [0.273, 0.468]	0.645 [0.573, 0.706]	−0.376 [−0.469, −0.274]	−0.419 [−0.508, −0.321]	−0.486 [−0.568, −0.395]	−0.107 [−0.217, 0.007]	−0.142 [−0.252, −0.029]	0.181 [0.069, 0.289]
TriPM Disinhibition	0.817	0.001	<0.001	1	0.632 [0.558, 0.695]	0.354 [0.251, 0.450]	0.605 [0.527, 0.672]	0.573 [0.491, 0.644]	−0.314 [−0.412, −0.208]	0.010 [−0.104, 0.123]	−0.224 [−0.329, −0.114]	−0.022 [−0.135, 0.091]	−0.149 [−0.258, −0.037]	0.328 [0.223, 0.426]
TriPM Total	0.394	<0.001	<0.001	<0.001	1	0.564 [0.482, 0.637]	0.319 [0.213, 0.417]	0.570 [0.488, 0.642]	−0.213 [−0.319, −0.103]	−0.333 [−0.430, −0.228]	−0.324 [−0.422, −0.219]	−0.097 [−0.208, 0.016]	−0.061 [−0.174, 0.052]	0.009 [−0.105, 0.122]
LSRP Primary	0.005	<0.001	<0.001	<0.001	<0.001	1	0.284 [0.176, 0.385]	0.860 [0.828, 0.887]	−0.234 [−0.338, −0.124]	−0.283 [−0.384, −0.175]	−0.314 [−0.413, −0.208]	−0.066 [−0.178, 0.048]	−0.066 [−0.178, 0.048]	0.140 [0.028, 0.250]
LSRP Secondary	0.229	<0.001	<0.001	<0.001	<0.001	<0.001	1	0.733 [0.675, 0.781]	−0.279 [−0.381, −0.171]	0.092 [−0.021, 0.204]	−0.157 [−0.266, −0.045]	0.036 [−0.078, 0.149]	−0.117 [−0.227, −0.004]	0.458 [0.363, 0.543]
LSRP Total	0.008	0.784	<0.001	<0.001	<0.001	<0.001	<0.001	1	−0.315 [−0.413, −0.209]	−0.152 [−0.261, −0.039]	−0.307 [−0.406, −0.200]	−0.028 [−0.141, 0.086]	−0.109 [−0.219, 0.005]	0.343 [0.239, 0.439]
QCAE Cognitive	0.906	<0.001	<0.001	<0.001	<0.001	<0.001	<0.001	<0.001	1	0.284 [0.177, 0.385]	0.874 [0.845, 0.899]	0.081 [−0.033, 0.192]	0.335 [0.230, 0.432]	−0.319 [−0.417, −0.213]
QCAE Affective	0.008	<0.001	<0.001	0.822	<0.001	<0.001	0.036	<0.001	<0.001	1	0.714 [0.653, 0.765]	0.103 [−0.010, 0.214]	−0.001 [−0.115, 0.112]	0.089 [−0.025, 0.200]
QCAE Total	0.153	0.950	<0.001	<0.001	<0.001	<0.001	<0.001	<0.001	<0.001	<0.001	1	0.111 [−0.002, 0.222]	0.244 [0.134, 0.348]	−0.188 [−0.295, −0.076]
BPQ Body Awareness	<0.001	0.113	0.016	0.612	0.027	0.134	0.415	0.530	0.068	0.019	0.012	1	0.204 [0.093, 0.310]	−0.030 [−0.143, 0.084]
IAS	0.067	0.002	0.001	<0.001	0.164	0.135	0.008	0.013	<0.001	0.976	<0.001	<0.001	1	−0.291 [−0.391, −0.184]
TAS	0.028	<0.001	<0.001	<0.001	0.844	0.001	<0.001	<0.001	<0.001	0.043	<0.001	0.500	<0.001	1

Above the diagonal: correlation coefficients and corresponding 99% confidence intervals; bellow the diagonal: *p*-values.

**Table 4:** Correlation comparison (Steiger’s Z-test for dependent correlations).

	**Z**	***p*-value**
*TriPM Boldness* and *QCAE Cognitive* vs. *TriPM Meanness* and *QCAE Cognitive*	10.974	<0.001
*TriPM Boldness* and *QCAE Cognitive* vs. *TriPM Disinhibition* and *QCAE Cognitive*	7.848	<0.001
*TriPM Disinhibition* and *QCAE Affective* vs. *TriPM Meanness* and *QCAE Affective*	10.546	<0.001
*TriPM Disinhibition* and *QCAE Affective* vs. *TriPM Boldness* and *QCAE Affective*	4.281	<0.001
*LSRP Primary* and *QCAE Cognitive* vs. *LSRP Secondary* and *QCAE Cognitive*	0.892	0.372
*LSRP Primary* and *QCAE Affective* vs. *LSRP Secondary* and *QCAE Affective*	−7.306	< 0.001
*TriPM Bldness* and *QCAE Cognitive* vs. *LSRP Primary* and *QCAE Cognitive*	7.827	<0.001
*TriPM Boldness* and *QCAE Cognitive* vs. *LSRP Secondary* and *QCAE Cognitive*	6.925	<0.001
*TriPM Disinhibition* and *QCAE Affective* vs. *LSRP Primary* and *QCAE Affective*	5.996	<0.001
*TriPM Disinhibition* and *QCAE AFFECTIVE* vs. *LSRP Secondary* and *QCAE Affective*	−2.093	0.036
*TriPM Boldness* and *IAS* vs. *TriPM Meanness* and *IAS*	5.089	<0.001
*TriPM Boldness* and *IAS* vs. *TriPM Disinhibition* and *IAS*	4.269	<0.001
*TriPM Disinhibition* and *BPQ* vs. *TriPM Meanness* and *BPQ*	1.969	0.049
*TriPM Disinhibition* and *BPQ* vs. *TriPM Boldness* and *BPQ*	0.720	0.471
*LSRP Primary* and *IAS* vs. *LSRP Secondary* and *IAS*	0.970	0.332
*LSRP Primary* and *BPQ* vs. *LSRP Secondary* and *BPQ*	−1.932	0.053
*TriPM Boldness* and *IAS* vs. *LSRP Primary* and *IAS*	3.594	<0.001
*TriPM Boldness* and *IAS* vs. *LSRP Secondary* and *IAS*	3.607	<0.001
*TriPM Disinhibition* and *BPQ* vs. *LSRP Primary* and *BPQ*	0.877	0.380
*TriPM Disinhibition* and *BPQ* vs. *LSRP Secondary* and *BPQ*	−1.478	0.140
*BPQ and QCAE Affective* vs. *BPQ* and *QCAE Cognitive*	0.418	0.676
*BPQ and QCAE Affective* vs. *IAS* and *QCAE Affective*	1.872	0.061
*IAS and QCAE Cognitive* vs. *IAS* and *QCAE Affective*	6.608	<0.001
*IAS and QCAE Cognitive* vs. *BPQ* and *QCAE Cognitive*	4.754	<0.001

**Table 5:** Partial correlations (controlling for alexithymia).

		TriPM Boldness	TriPM Meanness	TriPM Disinhibition	LSRP Primary	LSRP Secondary	QCAE Cognitive	QCAE Affective
QCAE Cognitive	*r_p_* and *99% CIs*	0.067 [−0.047, 0.179]	−0.341 [−0.438, −0.237]	−0.234 [−0.338, −0.123]	−0.202 [−0.308, −0.090]	−0.158 [−0.267, −0.046]	-	-
	*p*-value	0.128	<0.001	<0.001	<0.001	<0.001	-	-
QCAE Affective	*r_p_* and *99% CIs*	−0.256 [−0.359, −0.147]	−0.444 [−0.531, −0.348]	−0.020 [−0.134, 0.093]	−0.299 [−0.399, −0.193]	0.058 [−0.056, 0.171]	-	-
	*p*-value	<0.001	<0.001	0.643	<0.001	0.187	-	-
BPQ Body Awareness	*r_p_* and *99% CIs*	−0.090 [−202, 0.023]	−0.103 [−0.214, 0.011]	−0.013 [−0.127, 0.100]	−0.063 [−0.175, 0.051]	0.056 [−0.058, 0.168]	0.075 [−0.039, 0.187]	0.106 [−0.007, 0.217]
	*p*-value	0.040	0.020	0.762	0.157	0.206	0.089	0.016
IAS	*r_p_* and *99% CIs*	0.015 [−0.099, 0.128]	−0.095 [−0.206, 0.018]	−0.060 [−0.172, 0.054]	−0.026 [−0.139, 0.087]	0.019 [−0.094, 0.132]	0.267 [0.158. 0.369]	0.026 [−0.088, 0.139]
	*p-*value	0.741	0.031	0.177	0.550	0.662	<0.001	0.559

The triarchic phenotypes covaried as expected, with meanness and disinhibition largely correlated, *r* = 0.519, *p* < 0.001, while boldness displayed opposite associations with meanness, *r* = 0.234, *p* < 0.001, and, disinhibition, *r* = −0.142, *p* < 0.001. Primary and secondary psychopathy were positively correlated, *r* = 0.284, *p* < 0.001, as well as cognitive and affective empathy, *r* = 0.284, *p* < 0.001. Cognitive empathy, interoceptive accuracy, and all psychopathy subscales were associated with alexithymia scores (all *p* ≤ 0.001, *r* = [−0.414, 0.458]), reinforcing the need to control for this construct in the regression models. Here it is important to highlight that alexithymia was positively related to all psychopathy dimensions (all *p* ≤ 0.001, *r* = [0.140, 0.458]), except for the strong negative correlation with boldness, *r* = −0.414, *p* < 0.001.

Correlational analyses also indicated different empathy profiles across psychopathy dimensions. Within the triarchic model, meanness was associated with reduced cognitive, *r* = −0.376, *p* < 0.001, and affective empathy, *r* = −0.419, *p* < 0.001. Boldness displayed a small positive correlation with cognitive empathy, *r* = 0.190, *p* < 0.001, while presenting a medium negative association with affective empathy, *r* = −0.269, *p* < 0.001. Conversely, disinhibition was only negatively associated with cognitive empathy, *r* = −0.314, *p* < 0.001, while being unassociated with the affective domain, *r* = 0.010, *p* = 0.822. In the classical 2-factor model, cognitive empathy was negatively related with both primary, *r* = −0.234, *p* < 0.001, and secondary psychopathy, *r* = −0.283, *p* < 0.001, while affective empathy was only diminished within primary psychopathy traits, *r* = −0.279, *p* < 0.001. After controlling for alexithymia, all the significant psychopathy-empathy associations were retained (*p* < 0.001 for all), except for the relation between boldness and cognitive empathy, *r*_partial_ = 0.067, *p* = 0.128. Importantly, Steiger’s Z-tests for dependent correlations further reinforced the postulated distinct empathy profiles across psychopathy dimensions (see Table 4). The correlation of cognitive empathy with boldness was significantly different from the correlations of this empathy domain with the remaining triarchic phenotypes (meanness and disinhibition) as well as with primary and secondary psychopathy (*p* < 0.001 for all). Moreover, the disinhibition-affective empathy correlation was significantly divergent from the association of affective empathy with boldness, meanness, and primary psychopathy (*p* < 0.001 for all). Within classical factors, cognitive empathy was similarly correlated (as postulated) with both primary and secondary psychopathy, *Z* = 0.892, *p* = 0.372, despite the unexpected difference within the affective empathy domain, *Z* = −7.306, *p* < 0.001.

Regarding interoception measures, the dissociation between the IAS and BPQ has been reported elsewhere (Campos et al., 2021), but it is important to highlight the unexpected positive association between these measures, *r* = 0.204, *p* < 0.001. However, the IAS and BPQ were differentially associated with alexithymia, with a negative correlation for the IAS, *r* = −0.291, *p* < 0.001, while the BPQ did not display a significant correlation, *r* = −0.030, *p* = 0.500, suggesting that these instruments are indeed measuring different interoception-related constructs. Interoception measures were also differentially associated with empathy domains. A strong positive association was observed between cognitive empathy and interoceptive accuracy, *r* = 0.335, *p* < 0.001, even after controlling for alexithymia, *r*_partial_ = 0.267, *p* < 0.001. Contrastingly, interoceptive attention was not significantly associated with either cognitive, *r* = 0.081, *p* = 0.068, or affective empathy, *r* = 0.103, *p* = 0.019. Steiger’s Z-tests further reinforced these results, as the correlation of the IAS with cognitive empathy was significantly different from the correlation of this interoception measure with the affective empathy domain *Z* = 6.608, *p* < 0.001, and from the correlation of interoception attention with cognitive empathy, *Z* = 4.754, < 0.001. Finally, there were no significant associations between BPQ Body Awareness and any of the psychopathy scores (*p* > 0.01 for all), although several small correlations were found for the IAS, as boldness was positively associated with interoceptive accuracy, *r* = 0.133, *p* = 0.002, while meanness, disinhibition, and secondary psychopathy were negatively correlated with this construct, *p* < 0.008 for all, *r* = [−0.149, −0.117]. However, neither of these correlations remained significant after controlling for alexithymia, *p* > 0.031 for all, *r*_partial_ = [−0.095, 0.019].

Overall, the results from correlational analyses largely confirmed was hypothesis regarding the specific empathy profiles across psychopathy dimensions as conceptualized by the triarchic and classical 2-factor models. Similarly, interoception measures were also differentially associated with empathy domains as well as with alexithymia. Opposingly, interoceptive attention and accuracy do not seem to play a major role within psychopathy traits, particularly after controlling for alexithymia. Regardless, univariate results also indicated that multivariate models (controlling for sex, age, and alexithymia) should be implemented when evaluating the interplay between psychopathy, empathy, and interoception.

### 3.2. Hierarchical linear regression models

#### 3.2.1. Psychopathy dimensions and empathy domains

Table 6 includes detailed statistical findings regarding regression models (Models 1–4) examining whether psychopathy dimensions (triarchic phenotypes or classical factors—included in block 3) are differentially associated with either cognitive or affective empathy after controlling for sociodemographics (block 1) and alexithymia (block 2). For Model 1 (triarchic phenotypes and cognitive empathy), sociodemographics were not associated with QCAE Cognitive, *R^2^* = 0.006, *F*_(2, 512)_ = 1.629, *p* = 0.197, while adding alexithymia subsequently produced significant changes, Δ*R^2^* = 0.104, *F*_(1, 511)_ = 60.019, *p* < 0.001, being negatively associated with cognitive empathy, β = −0.325, *p* < 0.001. Triarchic phenotypes also contributed significantly to predicting cognitive empathy, Δ*R^2^* = 0.134, *F*_(3, 508)_ = 30.075, *p* < 0.001. Alexithymia was still a significant predictor after this final block, β = −0.155, *p* < 0.001, but boldness and meanness emerged as additional significant predictors, despite displaying opposite associations with cognitive empathy (β = 0.215, *p* < 0.001 and β = −0.384, *p* < 0.001, respectively). Replacing triarchic phenotypes for classical factors (Model 2) also significantly contributed to predicting cognitive empathy, Δ*R^2^* = 0.043, *F*_(2, 509)_ = 12.938, *p* < 0.001, as both primary and secondary psychopathy were negatively associated with this empathy domain (β = −0.160, *p* < 0.001 and β = −0.124, *p* = 0.009, respectively), despite alexithymia remaining a significant predictor as well, β = −0.246, *p* < 0.001.

**Table 6:** Hierarchical linear regression models examining psychopathy dimensions (triarchic phenotypes and classical factors) as predictors of empathy domains (cognitive and affective).

Independent variables	QCAE Cognitive Empathy		QCAE Affective Empathy	
Models	*Model 1 TriPM*	*Model 2 LSRP*	*Model 3 TriPM*	*Model 4 LSRP*
**Block 1: Demographics**				
Sex	−0.081		−0.292^**^	
Age (years)	0.011		−0.059	
*R^2^*	0.006		0.095	
*F*	1.629		27.010^**^	
**Block 2: Alexithymia**				
Sex	−0.090		−0.290^**^	
Age (years)	−0.019		−0.052	
TAS	−0.325^**^		0.071	
Δ*R^2^*	0.104		0.005	
*F*	60.019^**^		2.837	
**Block 3: Psychopathy**				
Sex	0.003	−0.037	−0.118^*^	−0.205^**^
Age (years)	−0.054	−0.048	−0.107^*^	−0.100^†^
TAS	−0.155^**^	−0.246^**^	0.065	0.042
TriPM Boldness	0.215^**^	-	−0.028	-
TriPM Meanness	−0.384^**^	-	−0.529^**^	-
TriPM Disinhibition	−0.033	-	0.281^**^	-
LSRP Primary	-	−0.160^**^	-	−0.289^**^
LSRP Secondary	-	−0.124^*^	-	0.150^*^

*n* = 515; standardized beta weights are presented for each predictor; ^*^p < 0.01; ^**^p < 0.001; ^†^*p* < 0.02. QCAE, Questionnaire of Cognitive and Affective Empathy; TAS, Toronto Alexithymia Scale; TriPM, Triarchic Psychopathy Measure; LSRP, Levenson Self-Report Psychopathy Scale.

Model 3 (triarchic phenotypes and affective empathy) revealed a significant contribution of sociodemographics, *R^2^* = 0.095, *F*_(2, 512)_ = 27.010, *p* < 0.001, driven by enhanced affective empathy in female subjects, β = −0.292, *p* < 0.001, while alexithymia was not a significant predictor, Δ*R^2^* = 0.005, *F*_(1, 511)_ = 2.837, *p* = 0.093. Triarchic phenotypes explained an additional 18.6% of the variance, Δ*R^2^* = 0.186, *F*_(3, 508)_ = 44.007, *p* < 0.001, with meanness being negatively related to affective empathy, β = −0.529, *p* < 0.001, while disinhibition displayed a positive association, β = 0.281, *p* < 0.001. After the last block, sex was still associated with affective empathy, β = −0.118, *p* = 0.007, while age emerged as a negative predictor, β = −0.107, *p* = 0.006. Inputting LSRP scores on the last block (Model 4) also produced significant changes in the model, Δ*R^2^* = 0.073, *F*_(2, 509)_ = 22.407, *p* < 0.001, driven by a similar pattern of results where primary and secondary psychopathy displayed opposite associations with affective empathy (β = −0.289, *p* < 0.001 and β = 0.150, *p* = 0.001, respectively). In this model, sex was still associated with this empathy domain, β = −0.205, *p* < 0.001, in contrast with age, β = −0.100, *p* = 0.017.

Altogether, results from the previously described models largely validate our predefined hypotheses regarding the interplay between psychopathy dimensions and empathy domains. Within the triarchic model, meanness displayed a broad empathy impairment. Conversely, adaptive boldness traits were associated with enhanced cognitive empathy, while disinhibition was positively related to affective empathy. Contrary to our hypotheses, boldness was unassociated with the affective domain and cognitive empathy was not negatively linked to disinhibition. Within the classical factors, there was indeed a broad empathy impairment in primary psychopathy, while secondary psychopathy was only negatively associated with cognitive empathy, although there was an unexpected positive association of the latter psychopathy factor with affective empathy. Finally, it is important to note that alexithymia was negatively associated with cognitive empathy (as postulated), while sex-related effects were only significant in the affective domain.

#### 3.2.2. Interoception measures and empathy domains

Additional models (Models 5 and 6) were implemented to evaluate the association of interoception measures (included in block 3) with cognitive and affective empathy—see Table 7 for full statistical findings. Importantly, the role of sociodemographics and alexithymia was also accounted for within these models, although the results from these control blocks will not be described here as they are similar to the findings previously reported for Models 1–4. Within Model 5, adding interoception measures produced significant changes, Δ*R^2^* = 0.067, *F*_(2, 509)_ = 20.681, *p* < 0.001, as IAS scores were positively associated with cognitive empathy, β = 0.271, *p* < 0.001, even though alexithymia was still a negative predictor of this empathy domain, β = −0.248, *p* < 0.001. In Model 6, including interoception scores as predictors did not significantly change the model, Δ*R^2^* = 0.004, *F*(2, 509) = 1.209, *p* = 0.299, although female subjects were still associated with larger affective empathy after this block, β = −0.285, *p* < 0.001. Results from these models partially support our hypotheses regarding the specific interaction between interoception and empathy domains, as the association concerning cognitive empathy and interoceptive accuracy was indeed established, despite the positive relation between affective empathy and interoceptive attention not being confirmed. As previously described, the interaction between interoception measures and empathy domains provides the landscape for the adequate interpretation of the putative role of interoception across psychopathy dimensions.

**Table 7:** Hierarchical linear regression models examining interoception measures (interoceptive attention and accuracy) as predictors of empathy domains (cognitive and affective).

Independent variables	QCAE Cognitive Empathy	QCAE Affective Empathy
Models	*Model 5*	*Model 6*
**Block 1: Demographics**		
Sex	−0.081	−0.292^**^
Age (years)	0.011	−0.059
*R^2^*	0.006	0.095
*F*	1.629	27.010^**^
**Block 2: Alexithymia**		
Sex	−0.090	−0.290^**^
Age (years)	−0.019	−0.052
TAS	−0.325^**^	0.071
Δ*R^2^*	0.104	0.005
*F*	60.019^**^	2.837
**Block 3: Interoception**		
Sex	−0.099^†^	−0.285^**^
Age (years)	−0.031	−0.046
TAS	−0.248^**^	0.083
BPQ Body Awareness	−0.001	0.052
IAS	0.271^**^	0.032
Δ*R^2^*	0.067	0.004
*F*	20.681^**^	1.209

*n* = 515; standardized beta weights are presented for each predictor; ^*^p < 0.01; ^**^p < 0.001; ^†^*p* < 0.02. QCAE, Questionnaire of Cognitive and Affective Empathy; TAS, Toronto Alexithymia Scale; BPQ, Body Perception Questionnaire; IAS, Interoceptive Accuracy Scale.

#### 3.2.3. Psychopathy dimensions and interoception measures

Regression models (Models 7–10) testing how psychopathy (triarchic phenotypes or classical dimensions—included in block 3) is associated with interoceptive attention or accuracy while controlling for sociodemographics (block 1) and alexithymia (block 2) are presented in Table 8. In Model 7 (triarchic phenotypes and interoceptive attention), sociodemographic variables significantly contributed to BPQ Body Awareness, *R^2^* = 0.037, *F*_(2, 512)_ = 9.755, *p* < 0.001, as age was a significant negative predictor, β = −0.135, *p* = 0.002. Opposingly, alexithymia, Δ*R^2^* = 0.002, *F*_(1, 511)_ = 1.230, *p* = 0.268, and triarchic phenotypes, Δ*R^2^* = 0.008, *F*_(3, 508)_ = 1.357, *p* = 0.255, did not produce significant changes in the model. Similarly, using classical factors instead of triarchic phenotypes (Model 8) on the last block did not produce significant changes in predicting interoceptive attention, Δ*R^2^* = 0.008, *F*_(2, 509)_ = 2.055, *p* = 0.129.

**Table 8:** Hierarchical linear regression models examining psychopathy dimensions (triarchic phenotypes and classical factors) as predictors of interoception measures (interoceptive attention and accuracy).

Independent variables	BPQ Body Awareness		IAS	
Models	*Model 7 TriPM*	*Model 8 LSRP*	*Model 9 TriPM*	*Model 10 LSRP*
**Block 1: Demographics**				
Sex	−0.112^†^		0.043	
Age (years)	−0.135^*^		0.072	
*R^2^*	0.037		0.008	
*F*	9.755^**^		2.145	
**Block 2: Alexithymia**				
Sex	−0.113^†^		0.036	
Age (years)	−0.139^*^		0.046	
TAS	−0.048		−0.285^**^	
Δ*R^2^*	0.002		0.080	
*F*	1.230		45.065**	
**Block 3: Psychopathy**				
Sex	−0.077	−0.092	0.081	0.048
Age (years)	−0.150^**^	−0.151^**^	0.033	0.039
TAS	−0.059	−0.077	−0.245^**^	−0.294^**^
TriPM Boldness	−0.023	-	0.029	-
TriPM Meanness	−0.098	-	−0.127	-
TriPM Disinhib.	0.060	-	−0.014	-
LSRP Primary	-	−0.073	-	−0.041
LSRP Secondary	-	0.084	-	0.031
Δ*R^2^*	0.008	0.008	0.014	0.002
*F*	1.357	2.055	2.625	0.486

*n* = 515; standardized beta weights are presented for each predictor; ^*^p < 0.01; ^**^p < 0.001; ^†^*p* < 0.02. Models 7 and 8 were implemented using wild bootstrapping due to heteroscedasticity. BPQ, Body Perception Questionnaire; IAS, Interoceptive Accuracy Scale; TAS, Toronto Alexithymia Scale; TriPM, Triarchic Psychopathy Measure; LSRP, Levenson Self-Report Psychopathy Scale.

In Model 9 (triarchic phenotypes and interoceptive accuracy) sociodemographics were not associated with IAS scores, *R^2^* = 0.008, *F*_(2, 512)_ = 2.145, *p* = 0.118, but including alexithymia significantly modified the model, *ΔR^2^* = 0.080, *F*_(1, 511)_ = 45.065, *p* < 0.001, due to a negative association with interoceptive accuracy, β = −0.285, *p* < 0.001. The last block with triarchic phenotypes did not produce significant changes in the model, Δ*R^2^* = 0.014, *F*_(3, 508)_ = 2.625, *p* = 0.050, and replacing these with classical psychopathy factors (Model 10) did not induce any specific contribution to interoceptive accuracy as well, Δ*R^2^* = 0.002, *F*_(2, 509)_ = 0.486, *p* = 0.616. Regardless, alexithymia remained a significant predictor of interoceptive accuracy after this last block in both models (β = −0.245, *p* < 0.001 and β = −0.294, *p* < 0.001, respectively).

Generally speaking, and widely contrary to our hypotheses, these models suggest that psychopathy dimensions are not associated with interoceptive attention and accuracy, regardless of whether the triarchic or classical 2-factor operationalization is used. Results regarding interoceptive attention are not as surprising within the context of the previously reported lack of association between affective empathy and this interoception-related construct. However, as interoceptive accuracy was related to cognitive empathy, it was feasible to expect that this construct would also play a role within the psychopathy personality structure. Finally, it is also important to highlight that alexithymia was only significantly associated with the interoceptive accuracy domain (as expected). The influence of sociodemographic variables on interoception measures was not congruent to our hypotheses, as there were no sex-related effects and age was only negatively associated with interoceptive attention.

### 3.3. Exploratory analyses

Based on the previously described findings and existing theoretical knowledge, exploratory path models were implemented to integrate the associations between psychopathy dimensions, empathy domains, interoception, and alexithymia. Theoretical frameworks, particularly introspection-centric simulation theory, have proposed that alexithymia can underlie reduced empathic processing (Goldman, 1992, 2006). The rationale is that an inability to adequately interpret our own affective states also interferes with our capacity to infer and/or share the emotional states of others (Bird and Viding, 2014). Valdespino et al. (2017) compiled behavioral and neural evidence exploring alexithymia as a transdiagnostic liability for empathy impairment across several psychopathological constructs, including psychopathic personality. There is evidence suggesting that empathy may mediate the link between alexithymia and psychopathy within the dark triad personality structure (Jonason and Krause, 2013), despite the caveat of this study not providing a multidimensional conceptualization of psychopathy. Burghart and Mier (2022) argued for the need to explore the putative mediating role of empathy within the alexithymia and psychopathy association, which could provide an important contribution to dissociate psychopathy dimensions. Complementarily, recent evidence from path models and network analysis has also suggested that alexithymia may be an important mediating bridge between interoception and empathy (Mul et al., 2018; Yang et al., 2022). Hence, existing evidence from path models combined with theoretical reasoning allows us to postulate that an indirect pathway, driven by alexithymia and/or empathy, may mediate the link between interoception and psychopathy. However, the specific interaction between psychopathy dimensions, empathy domains, and interoception measures is still completely unexplored.

In our confirmatory analyses, alexithymia and cognitive empathy were both related to interoceptive accuracy, while also displaying differential associations across psychopathy dimensions. Importantly, these specific connections between alexithymia and cognitive empathy were congruent within each psychopathy dimension (e.g., meanness was associated with higher alexithymia traits as well as with impaired cognitive empathy). Thus, it is feasible to postulate that interoceptive accuracy may underly variability in alexithymia and cognitive empathy, ultimately explaining how these constructs are differentially related to psychopathy dimensions. Path models were thus conducted to explore whether alexithymia and/or cognitive empathy act as mediators between interoceptive accuracy and psychopathy traits, with the additional upside of simultaneously accounting for the covariance of interoception and empathy domains, besides the shared variability within psychopathy dimensions already considered in regression analyses. Full statistical details for these models are provided in Supplementary material 2.

**Figure 1:**
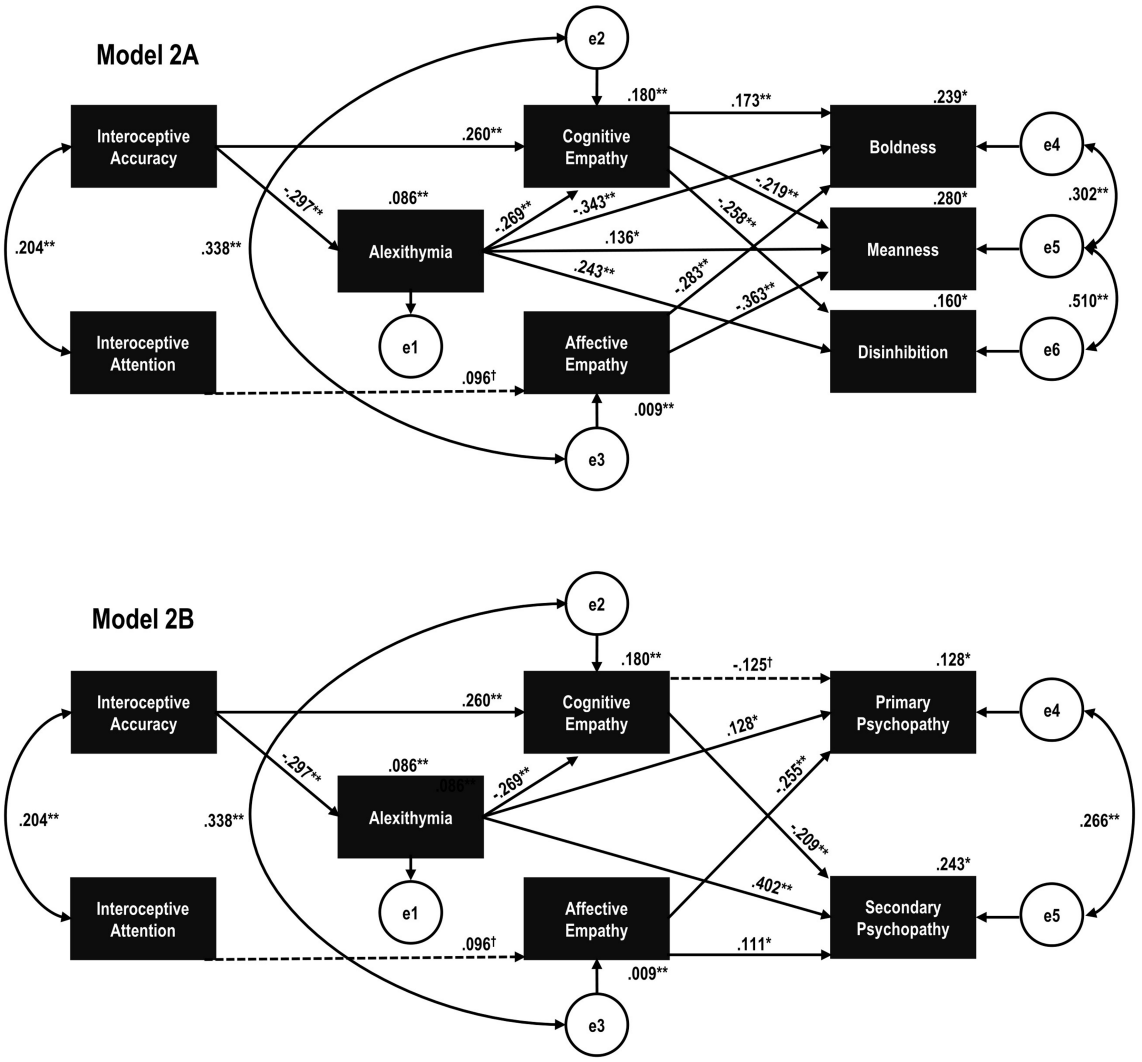
Final retained path models exploring alexithymia and/or cognitive empathy as mediators between interoception and psychopathy. (Model 2A) Triarchic phenotypes model. (Model 2B) Classical factors model. ^*^p < 0.001; ^**^p < 0.010; ^†^*p* < 0.020.

The first models implemented (Model 1A for triarchic phenotypes; Model 1B for classical factors) analyzed whether the previously postulated path directionality (interoception → alexithymia → empathy → psychopathy) provided an adequate alternative for framing our hypothesized preregistered associations between these constructs. Model 1A (triarchic phenotypes) displayed poor fit according to several statistics, χ^2^(6) = 89.085, *p* < 0.001, RMSEA = 0.164, RMSEA 90% CI = [0.135, 0.195], TLI = 0.533, CFI = 0.900, AIC = 149.085, BIC = 276.410. Similarly, despite being slightly better, Model 2A (classical 2-factors) also presented an inadequate fit to the data, χ^2^(4) = 26.542, *p* < 0.001, RMSEA = 0.105, RMSEA 90% CI = [0.069, 0.144], TLI = 0.743, CFI = 0.951, AIC = 74.542, BIC = 176.402. Modification indices suggested adding a direct effect between alexithymia and boldness in Model 1A as well the covariance between the IAS and BPQ Body Awareness within both models. These modifications were thus included, originating the final retained models (Figure 1) for both triarchic phenotypes (Model 2A) and classical factors (Model 2B). Model 2A displayed good fit according to most measures, χ^2^(4) = 5.079, *p* = 0.279, RMSEA = 0.023, RMSEA 90% CI = [0.000, 0.074], TLI = 0.991, CFI = 0.999, AIC = 69.079, BIC = 204.892, indicating the improved fitness in contrast to Model 1A. An equivalent improvement was observed for Model 2B, which also presented good fit accordingly to most statistics, χ^2^(3) = 4.670, *p* = 0.198, RMSEA = 0.033, RMSEA 90% CI = [0.000, 0.087], TLI = 0.975, CFI = 0.996; AIC = 54.670; BIC = 160.774.

Within the final model retained for triarchic phenotypes (Figure 1A - Model 2A), each dimension was still differentially associated with cognitive and affective empathy, despite some changes after also considering the covariance between empathy domains. More specifically, affective empathy deficits emerged in boldness (β = −0.283, *p* < 0.001), while disinhibition was now negatively associated with cognitive empathy (β = −0.258, *p* < 0.001) and no longer positively related to affective empathy (β = 0.061, *p* = 0.175). Alexithymia displayed significant and positive direct associations with meanness (β = 0.136, *p* = 0.003) and disinhibition (β = 0.243, *p* < 0.001), with negative effects observed for boldness (β = −0.343, *p* < 0.001) and cognitive empathy (β = −0.269, *p* < 0.001). Interoceptive accuracy was oppositely associated with cognitive empathy and alexithymia (β = 0.260, *p* < 0.001 and β = −0.297, *p* < 0.001, respectively). Conversely, there were no direct effects of interoception on triarchic phenotypes (*p > 0*.116 for all). Importantly, all indirect effects linking interoceptive accuracy to triarchic phenotypes *via* alexithymia and/or cognitive empathy were significant (all *p* < 0.002). This included a serial mediation effect linking interoceptive accuracy to triarchic phenotypes *via* alexithymia and cognitive empathy.

In the retained path model for classical psychopathy factors (Figure 1B - Model 2B), there were still two distinct empathy profiles for primary psychopathy (significant affective impairment, β = −0.255, *p* < 0.001, and marginally significant cognitive empathy deficits, β = −0.125, *p* = 0.013) and secondary psychopathy (negative association with cognitive empathy, β = −0.209, *p* < 0.001 and positive relation with the affective domain, β = 0.111, *p* = 0.006), although alexithymia was positively related to both factors (β = 0.128, *p* = 0.005 and β = 0.402, *p* < 0.001, respectively). However, as observed in the triarchic model, there was no direct association between interoception and either primary or secondary psychopathy traits (*p > 0*.186 for all). All indirect effects between interoceptive accuracy and classical psychopathy factors were also significant, as alexithymia and/or cognitive empathy were significant mediators (*p* < 0.009 for all).

Although somewhat unexpected, BPQ Body Awareness was marginally associated with affective empathy in both retained path models (β = 0.096, *p* = 0.010), and this empathy domain significantly mediated the link between interoceptive attention and all psychopathy scores (*p* < 0.009 for all), except for disinhibition (*p* = 0.105).

### 3.4. Control analyses

Excluding careless responders, outliers, and other confounders did not produce any major changes in the current findings (full outputs available at https://osf.io/zyf4e/). Univariate analyses were largely unaffected, although several small significant correlations were no longer significant (boldness with disinhibition, age with primary psychopathy and affective empathy, interoceptive accuracy with several psychopathy dimensions). Importantly, results pertaining to triarchic phenotypes remained unchanged in the regression and path models. Conversely, findings regarding classical psychopathy factors sustained some modifications. The negative association between both classical factors and cognitive empathy was no longer statistically significant (Model 2), although primary psychopathy was still negatively related to this empathy domain on the path model. Similarly, affective empathy was no longer significantly associated with secondary psychopathy (Model 4 and path model). Consequently, the indirect effects of interoceptive accuracy on primary psychopathy *via* cognitive empathy and/or alexithymia were not significant, as well as the mediation effect of affective empathy between interoceptive attention and primary psychopathy. It is also important to highlight that the marginal direct effects of interoceptive attention on affective empathy reached significance on all path models in the control analyses.

## 4. Discussion

Despite the historical and widely discussed link between psychopathy and empathy, evidence identifying distinct empathy profiles across psychopathy dimensions is still lacking. In particular, there are not many studies comparing what part empathy plays within competing conceptualizations of psychopathy, such as the classical 2-factors framework and the triarchic model. Moreover, and importantly, understanding the underlying mechanisms of empathy impairment (or absence of) within psychopathy traits can also provide important insights into the etiological pathways of this personality construct. Recent neurobehavioral models have argued for the importance of interoception in empathic processing, which can also open the door to exploring the role of inner body sensations within the realm of psychopathy. Hence, using theory-driven measurement frameworks for each construct, the current study aimed to examine the complex interplay between psychopathy dimensions, empathy domains, and interoception measures. Importantly, additional putative confounders that have been strongly associated with these constructs were also accounted for within the analytical approach, namely sociodemographics (sex and age) and alexithymia. The major findings of the current work will be discussed in subsequent sections.

### 4.1. Distinct empathy profiles across psychopathy dimensions

Our hypothesis-driven analyses suggest that the triarchic phenotypes and classical factors of psychopathy are differentially associated with cognitive and affective empathy, even when considering the covariance between psychopathy dimensions. Exploratory analyses with path models (additionally accounting for covariance within empathy domains) further refined these empathy profiles. Primary psychopathy was associated with a broader empathy impairment as expected (cognitive empathy nearing significance), while secondary psychopathy was linked to reduced cognitive empathy and, unexpectedly, to enhanced affective empathy. Within the triarchic model, meanness was associated with multidomain empathy deficits and disinhibition only displayed diminished cognitive empathy (as hypothesized), somewhat replicating empathy profiles from the two classical factors. In contrast, boldness displayed a unique pattern congruent with the expected results, with enhanced cognitive empathy despite reduced affective empathy scores.

Altogether, these results are highly aligned with previous meta-analytical evidence, further expanding existing knowledge by comparing empathy profiles across conceptual frameworks within the same community sample. First, affective psychopathy traits (contemplated within meanness and primary psychopathy) were associated with a broad empathy impairment, despite larger effects observed within the affective empathy domain. These results are widely consistent with previous meta-analyses, which highlighted callous-affective-meanness traits as the core dimension underlying empathy deficits in psychopathy (Northam and Dadds, 2020; Waller et al., 2020; Burghart and Mier, 2022; Campos et al., 2022). Disinhibition and secondary psychopathy, which encompass behavioral manifestations proximally linked to antisocial behavior, were negatively related to cognitive empathy. This was also largely expected based on meta-analytical evidence from subscales indexing these behavioral manifestations of psychopathy (Burghart and Mier, 2022; Campos et al., 2022) as well as from the link between cognitive empathy and antisocial outcomes (Miller and Eisenberg, 1988; Jolliffe and Farrington, 2004; van Langen et al., 2014). Surprisingly, secondary psychopathy was positively associated with affective empathy, even after accounting for the covariance between empathy domains in the path model. Despite this effect being small, it was still not congruent with previous meta-analytical evidence that reported a negligible albeit significant negative association between impulsive-antisocial traits and affective empathy (Campos et al., 2022). One putative explanation could be the specific affective empathy subscales proposed within the QCAE. For instance, Burghart and Mier (2022) reported that secondary psychopathy (as measured by the LSRP) is differentially associated with empathic concern (significant and moderate negative association) and personal distress (non-significant positive effective size). Similarly, our exploratory analyses with subscale scores (Supplemental material 1) also suggest that secondary psychopathy was only positively associated with the emotional contagion subscale. Regardless, it is important to highlight that LSRP Secondary displayed somewhat fragile internal consistency within our sample, thus limiting the interpretability of its association with affective empathy.

Finally, and most importantly, the current findings provide further evidence for boldness traits as an important additional dimension of the psychopathic personality structure, as recently reported in the recent meta-analysis (Campos et al., 2022). Despite sharing the affective empathy impairment observed within the meanness phenotype and primary psychopathy, boldness was positively associated with cognitive empathy. The boldness phenotype was developed to encompass low fear tendencies within the context of interpersonal behavior, such as persuasiveness and dominance (Patrick and Drislane, 2015). Even though primary psychopathy includes interpersonal traits, these focus on maladaptive characteristics stemming from the influential Hare’s conceptualization (Levenson et al., 1995). Hence, the current results reinforce that boldness additionally maps adaptive interpersonal expressions of psychopathy that rely on intact (or even enhanced) cognitive empathy to achieve effective social functioning, despite an underlying inability to share the emotional states of others, that is, impaired affective empathy (Hoppenbrouwers et al., 2016; Gao et al., 2020; Campos et al., 2022; Glenn et al., 2022).

### 4.2. The interplay between interoception, alexithymia, and empathy

Another key contribution of the current work was the differential associations of interoception measures with cognitive and affective empathy. More specifically, according to our hypotheses, interoceptive accuracy was significantly related to cognitive empathy, despite being unassociated with the affective domain. Despite conflicting findings in the field, a recent systematic review suggests that interoceptive accuracy is related to perspective-taking in emotional scenarios (Baiano et al., 2021). However, most existing studies only targeted performance-based cardiac interoceptive accuracy and often used a widely criticized heartbeat counting task (e.g., Brener and Ring, 2016; Corneille et al., 2020; Ferentzi et al., 2022). The current findings expand on previous evidence by reporting a positive association of interoception accuracy and cognitive empathy using self-report beliefs-based measures and after controlling for putative confounders such as sex, age, and alexithymia.

Importantly, within our sample, alexithymia was also negatively associated with both interoceptive accuracy and cognitive empathy. As previously discussed, alexithymia has been discussed as a contributing factor for empathy impairment as well as a putative mediator between interoception and empathy (Goldman, 1992, 2006; Bird and Viding, 2014; Valdespino et al., 2017). Evidence from network analyses suggested that enhanced interoception (broadly conceptualized) is concomitantly associated with improved empathic abilities and reduced alexithymia (Yang et al., 2022). Mul et al. (2018) reported that alexithymia mediated the association of specific interoception-related subscales with total empathy scores. The existing evidence is, however, largely unspecific, as it does not examine how exact theory-informed interoception and/or empathy domains play a role within these models. Hence, our results further contribute to this discussion, suggesting that this mediation effect may only emerge within the scope of interoceptive accuracy and cognitive empathy. That is, accurately perceiving our inner body information contributes to the effective understanding of our affective experiences, which consequently allows us to build adequate inferences about the emotional states of others.

Finally, our results suggest that interoception attention is not related to affective empathy, contrary to our hypothesis. The presumed link between interoceptive attention and affective empathy was driven by an embodied perspective regarding the vicarious experience of sharing the feelings of others (e.g., Goldman and de Vignemont, 2009; de Waal and Preston, 2017; Riečanský and Lamm, 2019). Presumably, observing someone experiencing any given emotional state (e.g., pain or disgust) activates physiological responses within our body, which would be more easily perceived and/or heavily weighted by subjects with an enhanced allocation of attentional resources to interoceptive stimuli. However, one can argue whether these lower-level processes of interoceptive attention are adequately captured when using self-report questionnaires to assess beliefs about interoception attention. Alternatively, implicit measures recording neural activity when subjects are required to focus their attention on interoceptive vs. exteroceptive stimuli may provide an interesting alternative to further examine the interplay between interoceptive attention and empathy (Ernst et al., 2013; Farb et al., 2013; Kuehn et al., 2016; Petzschner et al., 2019).

### 4.3. Interoception within psychopathy: Indirect effects driven by alexithymia and/or empathy

The final major contribution of the current work was exploring the association of self-reported interoceptive attention and accuracy with psychopathy dimensions. Neither of the interoception measures was associated with any psychopathy subscale after controlling for alexithymia, contrary to our hypotheses. Although no evidence existed until now assessing the association between interoception and triarchic phenotypes, previous results using the classical model display conflicting findings. Secondary psychopathy has been negatively related to interoceptive accuracy, as measured by heartbeat detection performance (Nentjes et al., 2013) as well as with specific interoceptive subscales indexing a construct that is closer to self-reported interoceptive attention (Lyons and Hughes, 2015). Conversely, Zwets et al. (2014) found no significant association of psychopathy with anger-specific bodily sensations, while a recent report also suggested that both classical psychopathy factors are unrelated to self-report and performance-based tasks of interoception (Lamoureux and Glenn, 2021).

Despite the absence of direct associations between interoception and psychopathy, we correctly predicted positive relations between alexithymia and several psychopathy scores (meanness, disinhibition, primary and secondary psychopathy), although there was also a strong unexpected and negative association with boldness. These findings, together with the previously described association between cognitive empathy and alexithymia, led us to conduct exploratory path models to investigate whether the link between interoception and psychopathy could be mediated by alexithymia and/or cognitive empathy. Our rationale was that interoceptive accuracy may underly variability in alexithymia and cognitive empathy, ultimately explaining how these constructs are differentially associated with psychopathy dimensions. Although these analyses were exploratory, significant indirect effects were indeed found between interoceptive accuracy and all psychopathy dimensions *via* either alexithymia, cognitive empathy, or both.

As previously described, existing theoretical proposals and empirical work have argued that alexithymia may underlie the empathy impairment typically associated with psychopathy traits (Krause et al., 2013; Bird and Viding, 2014; Valdespino et al., 2017). The current work further adds an additional layer to this discussion, with interoceptive processing emerging as a putative low-level mechanism underlying alexithymia and/or cognitive empathy. Without neglecting the exploratory nature of these findings, it is important to reason that there is neurobiological evidence supporting at least some degree of shared variance between these constructs. When discussing alexithymia as a transdiagnostic source of empathy impairment across clinical disorders, Valdespino et al. (2017) argued for the centrality of the insula within the co-occurrence of alexithymia and empathy impairments, including those observed in psychopathic personality. Importantly, the insula is also a core hub for interoceptive processing (Craig, 2009; Adolfi et al., 2017; Berntson and Khalsa, 2021) as well as a brain structure that has been implicated within the neurobiological and etiological pathways of psychopathy (Blair, 2013; Poeppl et al., 2019; Penagos-Corzo et al., 2022). Hence, the partial convergence between interoception, alexithymia, empathy, and psychopathy from a neuroanatomical standpoint could suggest an intricate (likely with some degree of causality) interaction between these constructs.

### 4.4. Limitations and recommendations for future studies

Despite the important contribution of the current work, several limitations and recommendations for future work should be addressed. The first major issue is the need to interpret the mediating effects stemming from the exploratory path models with caution, as these models were not postulated *a priori*. The direction of the associations between interoception, alexithymia, empathy, and psychopathy was proposed based on theoretical groundings as well as evidence stemming from less complex path models, but there is no strong empirical work to support the causality of the proposed interactions. Thereby, longitudinal studies or experimental work evaluating neural markers of interoceptive, emotional, and empathic processing would be ideal to further explore putative causal relations, despite the challenging nature of these endeavors.

Another important caveat is that within this study we did not examine the more fine-grained 4-facets model stemming from the classical 2-factors models (Neumann et al., 2007; Hare et al., 2018). This model proposed 4 correlated first-order factors without losing model fit, namely the interpersonal and affective facets (stemming from primary psychopathy) as well as the impulsive and antisocial facets (underlying secondary psychopathy). Hence, future studies should employ alternative instruments such as the Self-Report Psychopathy Scale (Paulhus et al., 2016), which includes a specific facet for maladaptive interpersonal traits (e.g., scamming people, pushing people to breaking point) as well as a more explicit antisocial facet (e.g., serious crime, carry weapons) that can provide valuable information to further understand empathy within the context of psychopathic personality (Campos et al., 2022).

Regarding empathy measurement, recent psychometric studies have queried whether the QCAE is an acceptable tool to index cognitive and affective empathy. Reniers et al. (2011) originally argued for a second-order structure of the QCAE, with cognitive empathy encompassing perspective-taking and online simulation as first-order factors, while the affective domain incorporated emotion contagion, proximal responsivity, and peripheral responsivity. Factor structure analyses as well as cross-domain correlations between these subscales have led several authors to question the broader cognitive and affective empathy domains within this questionnaire while favoring the first-order 5-factor oblique solution (Michaels et al., 2014; Myszkowski et al., 2017; Queirós et al., 2018; Di Girolamo et al., 2019; Liang et al., 2019; Gomez et al., 2022). However, these specific subscales can be somewhat debated as well due to their questionable or blatantly unacceptable internal consistency, in contrast to the acceptable-good reliability of broader cognitive and affective empathy scores. Regardless, we do still consider that the QCAE was the more adequate self-report measure available to index cognitive and affective empathy according to contemporary conceptual and neurobiological models. Concomitantly, it is obviously feasible to postulate that psychopathy, interoception, and alexithymia may be differentially related to lower-level empathy processes as those indexed by QCAE subscales or other alternative measures. Despite evidence still being recent and/or inconsistent, ongoing work has explored how specific (despite interlinked) second-level features of cognitive (e.g., inferring non-emotional vs. emotional mental states) and affective empathy (e.g., affective sharing, empathic concern, personal distress) are dissociable from a behavioral and neurobiological standpoint (Kalbe et al., 2010; Sebastian et al., 2012; Grynberg and López-Pérez, 2018; Grynberg and Konrath, 2020; Stevens and Taber, 2021). Hence, future studies could develop theory-driven hypotheses to examine how specific cognitive and affective empathy subprocesses interact with psychopathy, interoception, and alexithymia. Moreover, as recent evidence has questioned the convergence between self-perceived empathy vs. objective empathic abilities (Murphy and Lilienfeld, 2019; Sunahara et al., 2022), upcoming studies should examine whether the current pattern of results is replicated when using performance-based tasks or neural correlates of cognitive and affective empathy.

Another important drawback when contemplating the current findings concerning the role of interoception in psychopathy, empathy, and alexithymia is the somewhat questionable construct validity of interoception-related self-report measures (Desmedt et al., 2022). Recent evidence specifically indicated a lack of consistency among subjects when interpreting BPQ Body Awareness, as only 36.4% of participants considered that the questionnaire assessed interoceptive attention, while 30.4% interpreted it as pertaining to interoceptive accuracy (Gabriele et al., 2022). This could actually explain the positive association found between BPQ Body Awareness and IAS in the current dataset (Campos et al., 2021). Hence, there is still a need for future studies using novel and more reliable measures of interoception to assess its role in psychopathy, including not only self-report questionnaires (e.g., Interoception Attention Task; Gabriele et al., 2022) but also experimental tasks and neuronal correlates targeting different interoceptive pathways (e.g., Park and Blanke, 2019; Legrand et al., 2022; Nikolova et al., 2022).

Finally, it is important to highlight that some associations between empathy and classical psychopathy factors were no longer significant in the control analyses. This may be mainly due to small effect sizes between LSRP and QCAE scores (in contrast to triarchic phenotypes), making these associations more susceptible to the reduced power (lower sample size) in the control analyses.

### 4.5. Conclusion and main implications

Summing up, the current study provided a hypothesis-driven endeavor to examine the complex interplay between psychopathy dimensions, empathy domains, and interoception, using established frameworks to conceptualize each construct and controlling for important confounders such as sex, age, and alexithymia. The first major result, largely consistent with our hypotheses, was that distinct empathy profiles were observed across psychopathy dimensions. This clearly highlights the need for researchers in the field to contemplate the multidimensional nature of both constructs, as merely stating that empathy impairment is a hallmark of psychopathic personality can be nowadays seen as a widely non-specific and incomplete statement. Within the scope of psychopathy-empathy interplay, our findings indicate that boldness is associated with enhanced cognitive empathy, despite the co-occurring negative association with the affective domain. These results further reinforce the importance of the boldness phenotype within the constellation of psychopathy traits, as these adaptive interpersonal manifestations may help us to understand the longstanding emotion paradox of psychopathy as well as the more recently proposed profile for successful psychopathy.

Secondly, the current study also provided valuable evidence regarding the interplay between interoception and empathy, as within our sample a specific positive association between self-reported interoceptive accuracy and cognitive empathy was observed. Moreover, these constructs were also significantly related to alexithymia, providing evidence supporting emerging theoretical models of the neurobiological underpinnings of empathy, which argue that interoceptive processing contributes not only to perceiving our own emotional states but also to the ability to infer the feelings of others. Considering the transdiagnostic nature of alexithymia, these findings may also contribute to future work exploring how interoception may play a role in other empathy-related psychopathological constructs (e.g., autism, schizophrenia).

Lastly, in contrast to our hypotheses, self-perceived interoceptive attention and accuracy were not associated with either psychopathy dimension (triarchic or classical) after controlling for alexithymia. We thus proposed and exploratorily examined a theoretical mediation model where interoceptive accuracy could be indirectly linked to psychopathy *via* alexithymia and/or cognitive empathy. These indirect pathways were indeed observed and can provide a valuable venue for upcoming work aiming to explore the etiological pathways of empathy profiles across psychopathy dimensions. Furthermore, these findings may open the door for encompassing interoception-related strategies within behavioral interventions for emotional processing and empathy deficits in populations with high psychopathy traits. This may be useful when considering the modest efficacy and challenging nature of psychotherapeutic programs targeting psychopathy manifestations. Regardless, as our mediation effects were not planned under confirmatory testing, future preregistered hypothesis-driven studies should be designed to formally test this model while also accounting for other methodological limitations of the current study.

## Data availability statement

The datasets generated and analyzed for this study can be found in the Open Science Framework - https://osf.io/zyf4e/.

## Ethics statement

This study (involving human participants) was reviewed and approved by the Ethics Committee of the School of Health, Polytechnic University of Porto (reference E0048/2000, approved on 19 February 2020) and by the Data Protection Officer from the University of Porto (reference 2019101115001996, approved on 31 January 2020). Participants provided their informed consent (electronic form) to participate in this study.

## Author contributions

CC, NR, and FB were collaboratively responsible for study design and preregistration. The online survey was developed by CC with supervision from FB. Data collection and statistical analysis were conducted by CC. CC prepared the first draft of the manuscript which was then reviewed by NR and FB. All authors contributed to the article and approved the submitted version.

## Funding

This work was supported by a Ph.D. grant funded by the Portuguese Foundation for Science and Technology and the NORTE2020/FSE Program (SFRH/BD/136296/2018).

## Conflict of interest

The authors declare that the research was conducted in the absence of any commercial or financial relationships that could be construed as a potential conflict of interest.

## Publisher’s note

All claims expressed in this article are solely those of the authors and do not necessarily represent those of their affiliated organizations, or those of the publisher, the editors and the reviewers. Any product that may be evaluated in this article, or claim that may be made by its manufacturer, is not guaranteed or endorsed by the publisher.

## Footnotes

^1^TriPM meanness, disinhibition, and total scores displayed heterogeneity of variances.
