# Supplementary material for: Dissociating cognitive and affective empathy across psychopathy dimensions: The role of interoception and alexithymia
Source: Front Psychol. 2023 Jun 29;14:1082965. doi: 10.3389/fpsyg.2023.1082965 (PMC10345207; doi:10.3389/fpsyg.2023.1082965)
Supplement: Supplementary file 1 [file Data_Sheet_1.PDF]

Supplementary Material 1. Exploratory Correlational Analyses

**Correlations Between QCAE 5-Factor Subscales**				
QCAE_PerspectiveTaking - QCAE_OnlineSimulation	.428	<.001	.331	.516
QCAE_PerspectiveTaking - QCAE_EmotionalContagion	.099	.025	-.014	.210
QCAE_PerspectiveTaking - QCAE_ProximalResponsivity	.307	<.001	.200	.406
QCAE_PerspectiveTaking - QCAE_PeripheralResponsivity	.148	<.001	.035	.257
QCAE_OnlineSimulation - QCAE_EmotionalContagion	.101	.021	-.012	.212
QCAE_OnlineSimulation - QCAE_ProximalResponsivity	.313	<.001	.207	.412
QCAE_OnlineSimulation - QCAE_PeripheralResponsivity	.169	<.001	.056	.277
QCAE_EmotionalContagion - QCAE_ProximalResponsivity	.600	<.001	.522	.668
QCAE_EmotionalContagion - QCAE_PeripheralResponsivity	.270	<.001	.161	.372
QCAE_ProximalResponsivity - QCAE_PeripheralResponsivity	.364	<.001	.262	.459
**Correlations of QCAE 5-Factor Subscales with Higher-Level QCAE Scores**				
QCAE_PerspectiveTaking - QCAE_CognitiveEmpathy	.861	<.001	.829	.888
QCAE_PerspectiveTaking - QCAE_AffectiveEmpathy	.234	<.001	.124	.339
QCAE_PerspectiveTaking - QCAE_Total	.748	<.001	.693	.794
QCAE_OnlineSimulation - QCAE_CognitiveEmpathy	.828	<.001	.788	.860
QCAE_OnlineSimulation - QCAE_AffectiveEmpathy	.247	<.001	.137	.350
QCAE_OnlineSimulation - QCAE_Total	.730	<.001	.672	.779
QCAE_EmotionalContagion - QCAE_CognitiveEmpathy	.118	.007	.005	.229
QCAE_EmotionalContagion - QCAE_AffectiveEmpathy	.806	<.001	.762	.842
QCAE_EmotionalContagion - QCAE_Total	.494	<.001	.404	.576
QCAE_ProximalResponsivity - QCAE_CognitiveEmpathy	.367	<.001	.264	.461
QCAE_ProximalResponsivity - QCAE_AffectiveEmpathy	.836	<.001	.799	.867
QCAE_ProximalResponsivity - QCAE_Total	.691	<.001	.627	.746
QCAE_PeripheralResponsivity - QCAE_CognitiveEmpathy	.187	<.001	.075	.294
QCAE_PeripheralResponsivity - QCAE_AffectiveEmpathy	.696	<.001	.632	.750
QCAE_PeripheralResponsivity - QCAE_Total	.488	<.001	.397	.570
**Correlations of QCAE 5-Factor Subscales with Psychopathy Scores**				
QCAE_PerspectiveTaking - TriPM_Boldness	.255	<.001	.146	.358
QCAE_PerspectiveTaking - TriPM_Meanness	-.205	<.001	-.311	-.093
QCAE_PerspectiveTaking - TriPM_Disinhibition	-.180	<.001	-.288	-.068
QCAE_PerspectiveTaking - TriPM_TotalScore	-.037	.407	-.149	.077
QCAE_PerspectiveTaking - LSRP_Primary	-.126	.004	-.236	-.013
QCAE_PerspectiveTaking - LSRP_Secondary	-.157	<.001	-.265	-.044
QCAE_PerspectiveTaking - LSRP_TotalPsychopathy	-.173	<.001	-.281	-.061
QCAE_OnlineSimulation - TriPM_Boldness	.056	.203	-.057	.169
QCAE_OnlineSimulation - TriPM_Meanness	-.443	<.001	-.529	-.347

QCAE_OnlineSimulation - TriPM_Disinhibition	-.359	<.001	-.454	-.256
QCAE_OnlineSimulation - TriPM_TotalScore	-.339	<.001	-.436	-.235
QCAE_OnlineSimulation - LSRP_Primary	-.277	<.001	-.378	-.169
QCAE_OnlineSimulation - LSRP_Secondary	-.324	<.001	-.422	-.219
QCAE_OnlineSimulation - LSRP_TotalPsychopathy	-.369	<.001	-.463	-.267
QCAE_EmotionalContagion - TriPM_Boldness	-.336	<.001	-.432	-.231
QCAE_EmotionalContagion - TriPM_Meanness	-.300	<.001	-.400	-.193
QCAE_EmotionalContagion - TriPM_Disinhibition	.071	.109	-.043	.183
QCAE_EmotionalContagion - TriPM_TotalScore	-.287	<.001	-.388	-.179
QCAE_EmotionalContagion - LSRP_Primary	-.157	<.001	-.266	-.045
QCAE_EmotionalContagion - LSRP_Secondary	.186	<.001	.074	.293
QCAE_EmotionalContagion - LSRP_TotalPsychopathy	-.013	.770	-.126	.101
QCAE_ProximalResponsivity - TriPM_Boldness	-.193	<.001	-.300	-.082
QCAE_ProximalResponsivity - TriPM_Meanness	-.462	<.001	-.547	-.368
QCAE_ProximalResponsivity - TriPM_Disinhibition	-.068	.122	-.180	.045
QCAE_ProximalResponsivity - TriPM_TotalScore	-.347	<.001	-.443	-.243
QCAE_ProximalResponsivity - LSRP_Primary	-.317	<.001	-.415	-.211
QCAE_ProximalResponsivity - LSRP_Secondary	-.005	.914	-.118	.109
QCAE_ProximalResponsivity - LSRP_TotalPsychopathy	-.227	<.001	-.332	-.117
QCAE_PeripheralResponsivity - TriPM_Boldness	-.095	.031	-.206	.018
QCAE_PeripheralResponsivity - TriPM_Meanness	-.221	<.001	-.326	-.110
QCAE_PeripheralResponsivity - TriPM_Disinhibition	.017	.703	-.097	.130
QCAE_PeripheralResponsivity - TriPM_TotalScore	-.144	.001	-.253	-.031
QCAE_PeripheralResponsivity - LSRP_Primary	-.191	<.001	-.298	-.080
QCAE_PeripheralResponsivity - LSRP_Secondary	.029	.515	-.085	.142
QCAE_PeripheralResponsivity - LSRP_TotalPsychopathy	-.121	.006	-.231	-.007
**Correlations of QCAE 5-Factor Subscales with Interoception Scores**				
QCAE_PerspectiveTaking - BPQ_BodyAwareness	.085	.053	-.028	.197
QCAE_PerspectiveTaking - IAS_TotalScore	.347	<.001	.244	.443
QCAE_OnlineSimulation - BPQ_BodyAwareness	.049	.266	-.065	.162
QCAE_OnlineSimulation - IAS_TotalScore	.212	<.001	.101	.317
QCAE_EmotionalContagion - BPQ_BodyAwareness	.083	.060	-.031	.195
QCAE_EmotionalContagion - IAS_TotalScore	-.048	.282	-.160	.066
QCAE_ProximalResponsivity - BPQ_BodyAwareness	.018	.686	-.096	.131
QCAE_ProximalResponsivity - IAS_TotalScore	.064	.150	-.050	.176
QCAE_PeripheralResponsivity - BPQ_BodyAwareness	.139	.002	.026	.248
QCAE_PeripheralResponsivity - IAS_TotalScore	-.016	.718	-.129	.098
**Correlations of QCAE 5-Factor Subscales with Alexithymia Scores**				
QCAE_PerspectiveTaking - TAS_TotalScore	-.264	<.001	-.367	-.155
QCAE_PerspectiveTaking - TAS_IdentifyingFeelings	-.130	.003	-.240	-.017
QCAE_PerspectiveTaking - TAS_DescribingFeelings	-.217	<.001	-.322	-.106
QCAE_PerspectiveTaking - TAS_ExternallyOrientedThinking	-.278	<.001	-.379	-.170
QCAE_OnlineSimulation - TAS_TotalScore	-.276	<.001	-.377	-.168

QCAE_OnlineSimulation - TAS_IdentifyingFeelings	-.187	<.001	-.294	-.075
QCAE_OnlineSimulation - TAS_DescribingFeelings	-.181	<.001	-.288	-.069
QCAE_OnlineSimulation - TAS_ExternallyOrientedThinking	-.263	<.001	-.365	-.154
QCAE_EmotionalContagion - TAS_TotalScore	.244	<.001	.135	.348
QCAE_EmotionalContagion - TAS_IdentifyingFeelings	.340	<.001	.236	.437
QCAE_EmotionalContagion - TAS_DescribingFeelings	.180	<.001	.068	.288
QCAE_EmotionalContagion - TAS_ExternallyOrientedThinking	-.064	.148	-.176	.050
QCAE_ProximalResponsivity - TAS_TotalScore	.039	.373	-.074	.152
QCAE_ProximalResponsivity - TAS_IdentifyingFeelings	.173	<.001	.061	.281
QCAE_ProximalResponsivity - TAS_DescribingFeelings	.019	.663	-.094	.132
QCAE_ProximalResponsivity - TAS_ExternallyOrientedThinking	-.184	<.001	-.291	-.072
QCAE_PeripheralResponsivity - TAS_TotalScore	-.083	.059	-.195	.030
QCAE_PeripheralResponsivity - TAS_IdentifyingFeelings	.094	.032	-.019	.205
QCAE_PeripheralResponsivity - TAS_DescribingFeelings	-.038	.383	-.151	.075
QCAE_PeripheralResponsivity - TAS_ExternallyOrientedThinking	-.335	<.001	-.432	-.230
**Correlations of QCAE 5-Factor Subscales with Other Variables**				
QCAE_PerspectiveTaking - BPQ_AutonomicReactivity	-.100	.023	-.211	.014
QCAE_OnlineSimulation - BPQ_AutonomicReactivity	-.143	.001	-.252	-.030
QCAE_EmotionalContagion - BPQ_AutonomicReactivity	.171	<.001	.059	.279
QCAE_ProximalResponsivity - BPQ_AutonomicReactivity	.095	.032	-.019	.206
QCAE_PeripheralResponsivity - BPQ_AutonomicReactivity	.068	.125	-.046	.180
QCAE_PerspectiveTaking - Age	.032	.474	-.082	.144
QCAE_OnlineSimulation - Age	-.044	.317	-.157	.070
QCAE_EmotionalContagion - Age	-.088	.047	-.199	.026
QCAE_ProximalResponsivity - Age	-.008	.851	-.122	.105
QCAE_PeripheralResponsivity - Age	-.177	<.001	-.284	-.065
**Correlations of BPQ Autonomic Reactivity with Psychopathy Scores**				
BPQ_AutonomicReactivity - TriPM_Boldness	-.245	<.001	-.349	-.136
BPQ_AutonomicReactivity - TriPM_Meanness	.073	.097	-.040	.185
BPQ_AutonomicReactivity - TriPM_Disinhibition	.267	<.001	.158	.369
BPQ_AutonomicReactivity - TriPM_TotalScore	.025	.578	-.089	.138
BPQ_AutonomicReactivity - LSRP_Primary	.020	.658	-.094	.133
BPQ_AutonomicReactivity - LSRP_Secondary	.338	<.001	.233	.435
BPQ_AutonomicReactivity - LSRP_TotalPsychopathy	.193	<.001	.082	.300
**Correlations of BPQ Autonomic Reactivity with Empathy Scores**				
BPQ_AutonomicReactivity - QCAE_CognitiveEmpathy	-.142	.001	-.252	-.029
BPQ_AutonomicReactivity - QCAE_AffectiveEmpathy	.144	.001	.031	.253
BPQ_AutonomicReactivity - QCAE_Total	-.031	.481	-.144	.083
**Correlations of BPQ Autonomic Reactivity with Interoception Scores**				
BPQ_AutonomicReactivity - BPQ_BodyAwareness	.216	<.001	.105	.322
BPQ_AutonomicReactivity - IAS_TotalScore	-.267	<.001	-.369	-.158
**Correlations of BPQ Autonomic Reactivity with Alexithymia Scores**				
BPQ_AutonomicReactivity - TAS_TotalScore	.372	<.001	.270	.466
BPQ_AutonomicReactivity - TAS_IdentifyingFeelings	.456	<.001	.362	.542

BPQ_AutonomicReactivity - TAS_DescribingFeelings	.246	<.001	.137	.350
BPQ_AutonomicReactivity - TAS_ExternallyOrientedThinking	.033	.456	-.081	.146
**Correlations of BPQ Autonomic Reactivity with Other Variables**				
BPQ_AutonomicReactivity - Age	-.048	.281	-.160	.066
**Correlations Between TAS Subscales**				
TAS_IdentifyingFeelings - TAS_DescribingFeelings	.501	<.001	.411	.581
TAS_IdentifyingFeelings - TAS_ExternallyOrientedThinking	.159	<.001	.046	.267
TAS_DescribingFeelings - TAS_ExternallyOrientedThinking	.246	<.001	.136	.349
**Correlations of TAS Subscales with TAS Total Score**				
TAS_IdentifyingFeelings - TAS_TotalScore	.831	<.001	.792	.863
TAS_DescribingFeelings - TAS_TotalScore	.793	<.001	.747	.831
TAS_ExternallyOrientedThinking - TAS_TotalScore	.551	<.001	.467	.626
**Correlations of TAS Subscales with Psychopathy Scores**				
TAS_IdentifyingFeelings - TriPM_Boldness	-.382	<.001	-.475	-.281
TAS_IdentifyingFeelings - TriPM_Meanness	.089	.044	-.025	.200
TAS_IdentifyingFeelings - TriPM_Disinhibition	.367	<.001	.265	.461
TAS_IdentifyingFeelings - TriPM_TotalScore	.004	.929	-.109	.117
TAS_IdentifyingFeelings - LSRP_Primary	.041	.354	-.073	.154
TAS_IdentifyingFeelings - LSRP_Secondary	.485	<.001	.394	.567
TAS_IdentifyingFeelings - LSRP_TotalPsychopathy	.287	<.001	.179	.388
TAS_DescribingFeelings - TriPM_Boldness	-.358	<.001	-.453	-.255
TAS_DescribingFeelings - TriPM_Meanness	.090	.040	-.023	.202
TAS_DescribingFeelings - TriPM_Disinhibition	.129	.003	.016	.239
TAS_DescribingFeelings - TriPM_TotalScore	-.096	.029	-.207	.017
TAS_DescribingFeelings - LSRP_Primary	.032	.469	-.082	.145
TAS_DescribingFeelings - LSRP_Secondary	.273	<.001	.165	.375
TAS_DescribingFeelings - LSRP_TotalPsychopathy	.168	<.001	.056	.276
TAS_ExternallyOrientedThinking - TriPM_Boldness	-.134	.002	-.244	-.021
TAS_ExternallyOrientedThinking - TriPM_Meanness	.260	<.001	.151	.363
TAS_ExternallyOrientedThinking - TriPM_Disinhibition	.187	<.001	.076	.294
TAS_ExternallyOrientedThinking - TriPM_TotalScore	.132	.003	.019	.242
TAS_ExternallyOrientedThinking - LSRP_Primary	.290	<.001	.183	.391
TAS_ExternallyOrientedThinking - LSRP_Secondary	.194	<.001	.082	.300
TAS_ExternallyOrientedThinking - LSRP_TotalPsychopathy	.309	<.001	.203	.408
**Correlations of TAS Subscales with Empathy Scores**				
TAS_IdentifyingFeelings - QCAE_CognitiveEmpathy	-.186	<.001	-.293	-.074
TAS_IdentifyingFeelings - QCAE_AffectiveEmpathy	.262	<.001	.153	.365
TAS_IdentifyingFeelings - QCAE_Total	-.003	.946	-.116	.110
TAS_DescribingFeelings - QCAE_CognitiveEmpathy	-.236	<.001	-.340	-.126
TAS_DescribingFeelings - QCAE_AffectiveEmpathy	.071	.107	-.043	.183
TAS_DescribingFeelings - QCAE_Total	-.136	.002	-.246	-.023
TAS_ExternallyOrientedThinking - QCAE_CognitiveEmpathy	-.320	<.001	-.418	-.215
TAS_ExternallyOrientedThinking - QCAE_AffectiveEmpathy	-.247	<.001	-.351	-.138

TAS_ExternallyOrientedThinking - QCAE_Total	-.359	<.001	-.454	-.256
**Correlations of TAS Subscales with Interoception Scores**				
TAS_IdentifyingFeelings - BPQ_BodyAwareness	-.003	.950	-.116	.111
TAS_IdentifyingFeelings - IAS_TotalScore	-.296	<.001	-.396	-.189
TAS_DescribingFeelings - BPQ_BodyAwareness	.074	.095	-.040	.186
TAS_DescribingFeelings - IAS_TotalScore	-.180	<.001	-.287	-.068
TAS_ExternallyOrientedThinking - BPQ_BodyAwareness	-.166	<.001	-.274	-.054
TAS_ExternallyOrientedThinking - IAS_TotalScore	-.135	.002	-.245	-.022
**Correlations of TAS Subscales with Other Variables**				
TAS_IdentifyingFeelings - Age	-.133	.003	-.242	-.020
TAS_DescribingFeelings - Age	-.197	<.001	-.304	-.086
TAS_ExternallyOrientedThinking - Age	.171	<.001	.058	.279
