# Supplementary material for: Dissociating cognitive and affective empathy across psychopathy dimensions: The role of interoception and alexithymia
Source: Front Psychol. 2023 Jun 29;14:1082965. doi: 10.3389/fpsyg.2023.1082965 (PMC10345207; doi:10.3389/fpsyg.2023.1082965)
Supplement: Supplementary file 2 [file Data_Sheet_2.PDF]

Supplementary Material 2. Path Models

The initially implemented path models contemplated the hypothesized significant associations described in the study registration (https://osf.io/5jhcw/). The directionality of associations was proposed following the results stemming from hypothesis-driven analyses and theoretical knowledge. The rationale was that interoceptive accuracy may underly variability in alexithymia and cognitive empathy, ultimately explaining how these constructs are differentially related to psychopathy dimensions.

Direct and indirect effects were tested using path analysis in SPSS Amos v28. These models were estimated using maximum likelihood estimation and bootstrapping with 5000 resamples. Effects were thus computed with 99% bias-corrected confidence intervals (significance threshold .01). Univariate normality was assessed using a skewness (less than |2.0|) and kurtosis (less than |9.0|) threshold. Multivariate normality was evaluated using multivariate kurtosis (values > 5 indicative of departure from normality). Multivariate outliers were identified using Mahalanobis distance. The following indicators were used to describe the absolute model fit: chi-squared goodness-of-fit statistic (significance); Comparative Fit Index (CFI; ≥ .90 acceptable fit; ≥ .95 good fit); Tucker Lewis Index (TLI; ≥ .90 acceptable fit; ≥ .95 good fit); Root Mean Square Error of Approximation (RMSEA; ≤ .08 acceptable fit; ≤ .06 with 90% CIs ≤ .10 good fit). Relative model fit (model comparison) was assessed using the Akaike Information Criterion (AIC) and Bayesian Information Criterion (BIC) - lower values indicating better fit.

The graphical representation and main results of interest (model fit, direct effects, correlations, squared multiple correlations, specific indirect effects) from these models are presented below. Additional information regarding the presented models (assumption testing, total effects, etc) can be consulted within the full outputs at https://osf.io/zyf4e/.

Model 1A. Hypothesis-Driven Model for Triarchic Phenotypes

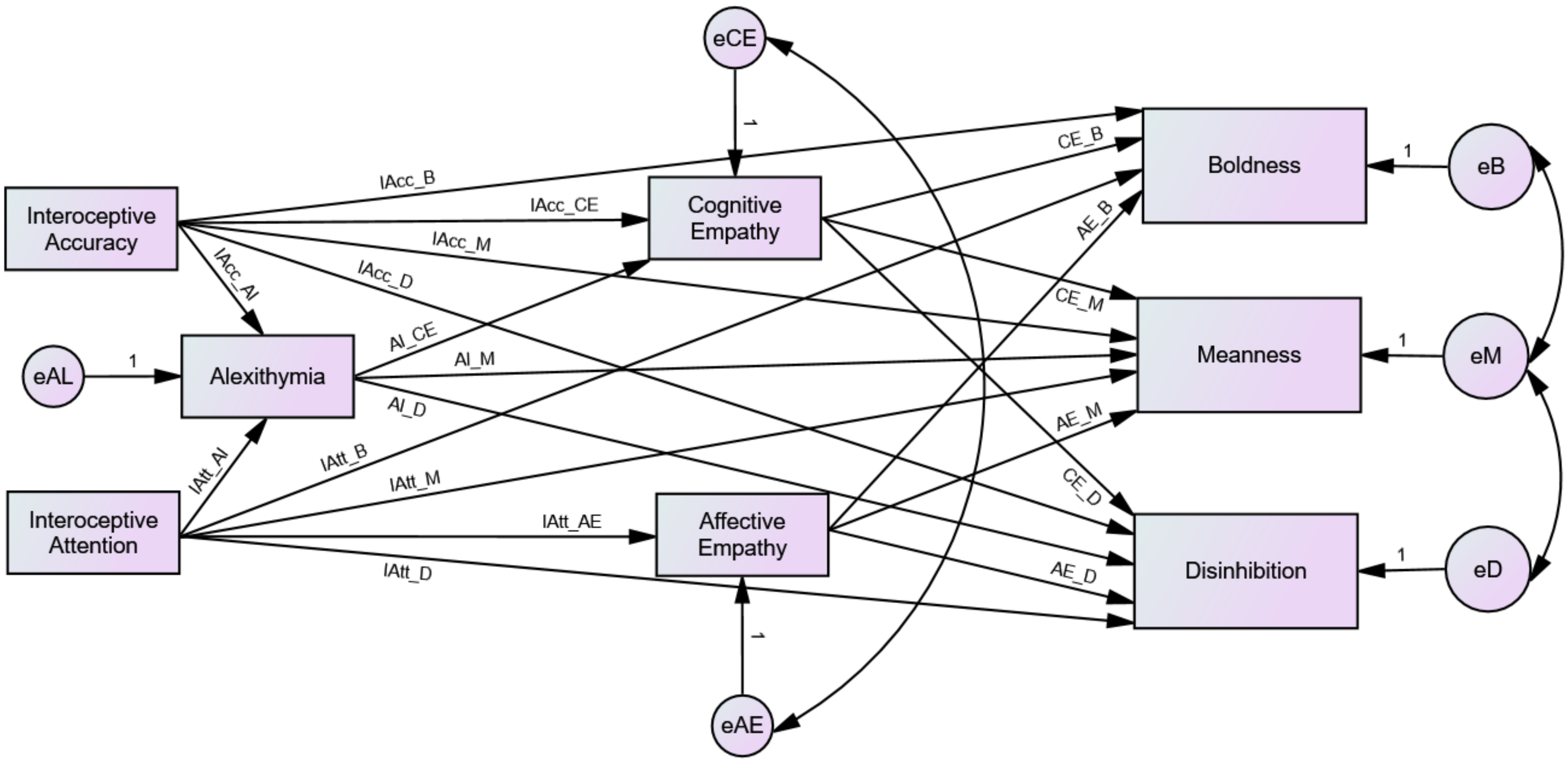

Goodness of fit statistics

$\chi^2(6) = 89.085$; $p < .001$; RMSEA = .164; RMSEA 90% CI = [.135, .195]; TLI = .533; CFI = .900; AIC = 149.085; BIC = 276.410

Table 1. *Direct effects, correlations and squared multiple correlations for Model 1A*

**Standardized Direct Effects (Regression Weights)**			**Estimate**	**99% CI Lower Limit**	**99% CI Upper Limit**	***p***
TAS_TotalScore	<---	BPQ_BodyAwareness	.031	-.074	.133	.456
TAS_TotalScore	<---	IAS_TotalScore	-.297	-.403	-.183	<.001
QCAE_CognitiveEmpathy	<---	IAS_TotalScore	.260	.156	.361	<.001
QCAE_AffectiveEmpathy	<---	BPQ_BodyAwareness	.096	.000	.195	.010
QCAE_CognitiveEmpathy	<---	TAS_TotalScore	-.269	-.369	-.160	<.001
TriPM_Disinhibition	<---	QCAE_CognitiveEmpathy	-.258	-.378	-.124	<.001
TriPM_Boldness	<---	IAS_TotalScore	.055	-.065	.176	.237
TriPM_Meanness	<---	QCAE_AffectiveEmpathy	-.372	-.459	-.275	<.001
TriPM_Meanness	<---	TAS_TotalScore	.232	.125	.326	<.001
TriPM_Disinhibition	<---	TAS_TotalScore	.243	.132	.358	<.001
TriPM_Disinhibition	<---	IAS_TotalScore	.008	-.109	.117	.895
TriPM_Boldness	<---	QCAE_CognitiveEmpathy	.276	.153	.391	<.001
TriPM_Boldness	<---	BPQ_BodyAwareness	-.068	-.179	.045	.109
TriPM_Meanness	<---	IAS_TotalScore	-.002	-.113	.110	.953
TriPM_Meanness	<---	BPQ_BodyAwareness	-.044	-.139	.052	.275
TriPM_Meanness	<---	QCAE_CognitiveEmpathy	-.184	-.302	-.063	<.001
TriPM_Boldness	<---	QCAE_AffectiveEmpathy	-.339	-.445	-.226	<.001
TriPM_Disinhibition	<---	QCAE_AffectiveEmpathy	.061	-.056	.173	.175
TriPM_Disinhibition	<---	BPQ_BodyAwareness	-.002	-.103	.102	.975
**Correlations**			**Estimate**	**99% CI Lower Limit**	**99% CI Upper Limit**	***p***
eCE	<-->	eAE	.338	.230	.440	<.001
eM	<-->	eB	.319	.218	.417	<.001
eM	<-->	eD	.507	.393	.602	<.001
**Squared Multiple Correlations**			**Estimate**	**99% CI Lower Limit**	**99% CI Upper Limit**	***p***
		TAS_TotalScore	.089	.034	.161	<.001
		QCAE_AffectiveEmpathy	.009	.000	.038	<.001
		QCAE_CognitiveEmpathy	.181	.104	.268	<.001
		TriPM_Boldness	.157	.081	.232	.002
		TriPM_Disinhibition	.161	.090	.232	.002
		TriPM_Meanness	.302	.205	.381	.002

Table 2. *Specific Indirect Effects for Model 1A*

Specific Indirect Effects (Unstandardized Weights)	Estimate	99% CI Lower Limit	99% CI Upper Limit	*p*
Interoceptive Accuracy --->Boldness Via Cognitive Empathy	.053	.028	.090	<.001
Interoceptive Accuracy ---> Meanness Via Cognitive Empathy	-.030	-.057	-.011	<.001
Interoceptive Accuracy ---> Disinhibition Via Cognitive Empathy	-.043	-.075	-.020	<.001
Interoceptive Accuracy ---> Meanness Via Alexithymia	-.043	-.072	-.020	<.001
Interoceptive Accuracy ---> Disinhibition Via Alexithymia	-.046	-.080	-.023	<.001
Interoceptive Accuracy ---> Boldness Via Alexithymia & Cognitive Empathy	.016	.007	.034	<.001
Interoceptive Accuracy ---> Meanness Via Alexithymia & Cognitive Empathy	-.009	-.019	-.003	<.001
Interoceptive Accuracy ---> Disinhibition Via Alexithymia & Cognitive Empathy	-.013	-.027	-.005	<.001
Interoceptive Attention ---> Boldness Via Affective Empathy	-.012	-.027	.000	.008
Interoceptive Attention ---> Meanness Via Affective Empathy	-.011	-.024	.000	.009
Interoceptive Attention ---> Disinhibition Via Affective Empathy	.002	-.001	.008	.105
Interoceptive Attention ---> Meanness Via Alexithymia	.002	-.005	.010	.435
Interoceptive Attention ---> Disinhibition Via Alexithymia	.002	-.006	.011	.428
Interoceptive Attention ---> Boldness Via Alexithymia & Cognitive Empathy	-.001	-.005	.002	.396
Interoceptive Attention ---> Meanness Via Alexithymia & Cognitive Empathy	.000	-.001	.003	.383
Interoceptive Attention ---> Disinhibition Via Alexithymia & Cognitive Empathy	.001	-.002	.004	.392
Alexithymia ---> Boldness Via Cognitive Empathy	-.054	-.096	-.023	<.001
Alexithymia ---> Meanness Via Cognitive Empathy	.030	.012	.055	<.001
Alexithymia ---> Disinhibition Via Cognitive Empathy	.043	.019	.076	<.001
Interoceptive Accuracy ---> Cognitive Empathy Via Alexithymia	.054	.027	.090	<.001

Modification indices: Covariances (IAS and BPQ Body Awareness; eB and eAL; eB and eCE); Direct Effects (TAS_TotalScore ---> TriPM_Boldness)

The covariance between IAS and BPQ Body Awareness and the direct effect from TAS_TotalScore to TriPM_Boldness were included in model 2A.

Model 2A. Hypothesis-Driven Model Plus Selected Modification Indices for Triarchic Phenotypes

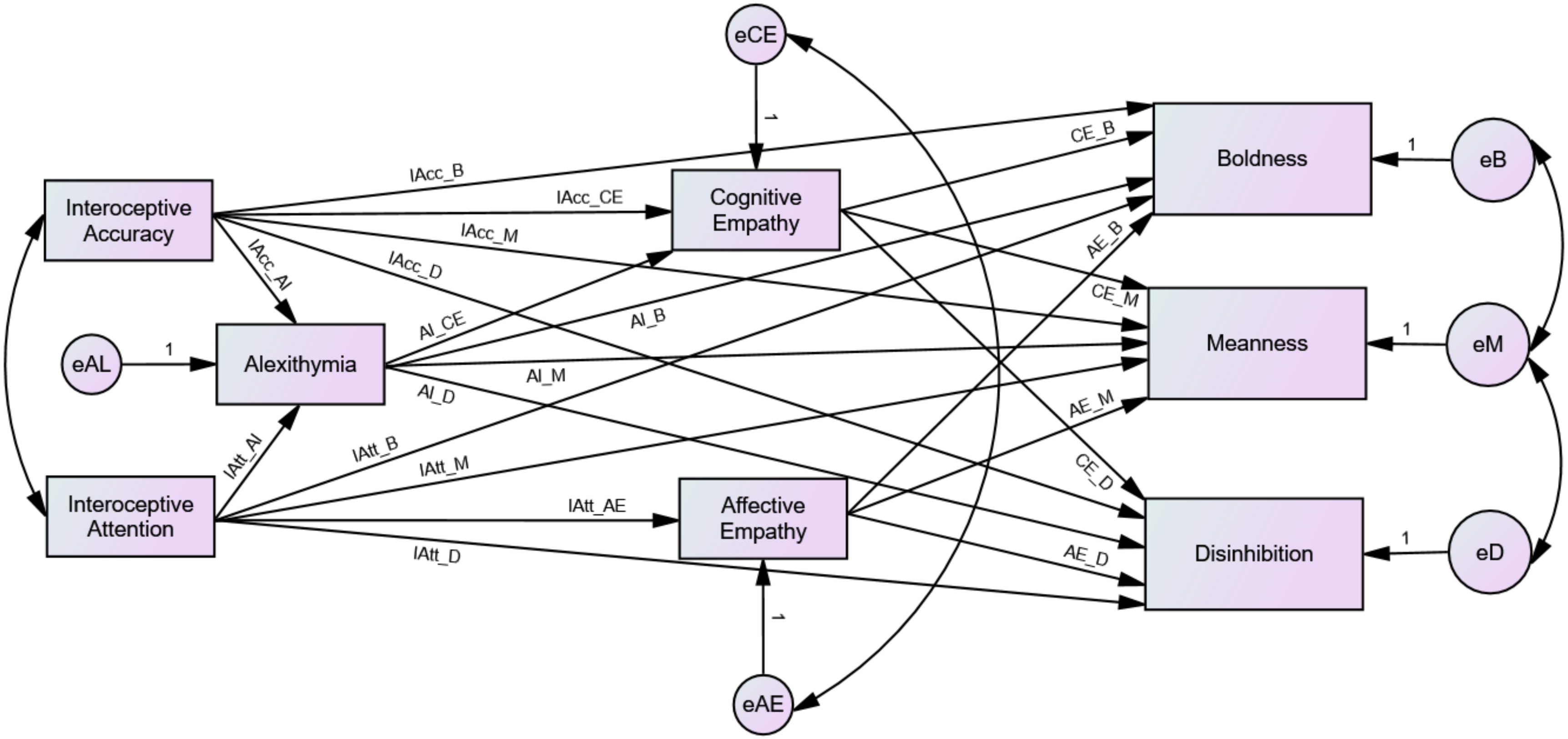

Goodness of fit statistics

$\chi^2(4) = 5.079$; $p = .279$; RMSEA = .023; RMSEA 90% CI = [.000, .074]; TLI = .991; CFI = .999; AIC = 69.079; BIC = 204.892

Table 3. *Direct effects, correlations and squared multiple correlations for Model 2A*

**Standardized Direct Effects (Regression Weights)**			**Estimate**	**99% CI Lower Limit**	**99% CI Upper Limit**	***p***
TAS_TotalScore	<---	BPQ_BodyAwareness	.031	-.074	.134	.456
TAS_TotalScore	<---	IAS_TotalScore	-.297	-.404	-.183	<.001
QCAE_CognitiveEmpathy	<---	IAS_TotalScore	.260	.156	.361	<.001
QCAE_AffectiveEmpathy	<---	BPQ_BodyAwareness	.096	.000	.195	.010
QCAE_CognitiveEmpathy	<---	TAS_TotalScore	-.269	-.368	-.159	<.001
TriPM_Disinhibition	<---	QCAE_CognitiveEmpathy	-.258	-.377	-.124	<.001
TriPM_Boldness	<---	IAS_TotalScore	-.010	-.120	.098	.832
TriPM_Meanness	<---	QCAE_AffectiveEmpathy	-.363	-.452	-.266	<.001
TriPM_Meanness	<---	TAS_TotalScore	.136	.027	.239	.003
TriPM_Disinhibition	<---	TAS_TotalScore	.243	.133	.358	<.001
TriPM_Disinhibition	<---	IAS_TotalScore	.008	-.109	.117	.894
TriPM_Boldness	<---	QCAE_CognitiveEmpathy	.173	.052	.287	<.001
TriPM_Meanness	<---	IAS_TotalScore	-.021	-.132	.093	.633
TriPM_Meanness	<---	BPQ_BodyAwareness	-.043	-.140	.057	.295
TriPM_Meanness	<---	QCAE_CognitiveEmpathy	-.219	-.336	-.103	<.001
TriPM_Disinhibition	<---	QCAE_AffectiveEmpathy	.061	-.056	.173	.175
TriPM_Disinhibition	<---	BPQ_BodyAwareness	-.002	-.103	.103	.975
TriPM_Boldness	<---	QCAE_AffectiveEmpathy	-.283	-.387	-.165	<.001
TriPM_Boldness	<---	TAS_TotalScore	-.343	-.448	-.236	<.001
TriPM_Boldness	<---	BPQ_BodyAwareness	-.063	-.166	.045	.116
**Correlations**			**Estimate**	**99% CI Lower Limit**	**99% CI Upper Limit**	***p***
IAS_TotalScore	<-->	BPQ_BodyAwareness	.204	.082	.307	<.001
eCE	<-->	eAE	.338	.230	.440	<.001
eM	<-->	eB	.302	.205	.395	<.001
eM	<-->	eD	.510	.397	.604	<.001
**Squared Multiple Correlations**			**Estimate**	**99% CI Lower Limit**	**99% CI Upper Limit**	***p***
		TAS_TotalScore	.086	.031	.155	<.001
		QCAE_AffectiveEmpathy	.009	.000	.038	<.001
		QCAE_CognitiveEmpathy	.180	.102	.265	<.001
		TriPM_Boldness	.239	.154	.320	.002
		TriPM_Disinhibition	.160	.089	.231	.002
		TriPM_Meanness	.280	.186	.361	.002

Table 4. *Specific Indirect Effects for Model 2A*

**Specific Indirect Effects (Unstandardized Weights)**	**Estimate**	**99% CI Lower Limit**	**99% CI Upper Limit**	***p***
Interoceptive Accuracy --->Boldness Via Cognitive Empathy	.033	.011	.063	<.001
Interoceptive Accuracy ---> Meanness Via Cognitive Empathy	-.035	-.063	-.016	<.001
Interoceptive Accuracy ---> Disinhibition Via Cognitive Empathy	-.043	-.075	-.020	<.001
Interoceptive Accuracy ---> Boldness Via Alexithymia	.075	.041	.115	<.001
Interoceptive Accuracy ---> Meanness Via Alexithymia	-.025	-.050	-.005	.002
Interoceptive Accuracy ---> Disinhibition Via Alexithymia	-.046	-.080	-.023	<.001
Interoceptive Accuracy ---> Boldness Via Alexithymia & Cognitive Empathy	.010	.003	.023	<.001
Interoceptive Accuracy ---> Meanness Via Alexithymia & Cognitive Empathy	-.011	-.021	-.004	<.001
Interoceptive Accuracy ---> Disinhibition Via Alexithymia & Cognitive Empathy	-.013	-.027	-.005	<.001
Interoceptive Attention ---> Boldness Via Affective Empathy	-.010	-.023	.000	.008
Interoceptive Attention ---> Meanness Via Affective Empathy	-.010	-.023	.000	.009
Interoceptive Attention ---> Disinhibition Via Affective Empathy	.002	-.001	.008	.105
Interoceptive Attention ---> Boldness Via Alexithymia	-.004	-.017	.009	.447
Interoceptive Attention ---> Meanness Via Alexithymia	.001	-.003	.007	.368
Interoceptive Attention ---> Disinhibition Via Alexithymia	.002	-.006	.011	.428
Interoceptive Attention ---> Boldness Via Alexithymia & Cognitive Empathy	-.001	-.003	.001	.367
Interoceptive Attention ---> Meanness Via Alexithymia & Cognitive Empathy	.001	-.001	.003	.400
Interoceptive Attention ---> Disinhibition Via Alexithymia & Cognitive Empathy	.001	-.002	.004	.392
Alexithymia ---> Boldness Via Cognitive Empathy	-.033	-.067	-.010	<.001
Alexithymia ---> Meanness Via Cognitive Empathy	.035	.016	.061	<.001
Alexithymia ---> Disinhibition Via Cognitive Empathy	.043	.019	.076	<.001
Interoceptive Accuracy ---> Cognitive Empathy Via Alexithymia	.054	.027	.090	<.001

Modification indices: None

Model 1B. Hypothesis-Driven Model for Classical Factors

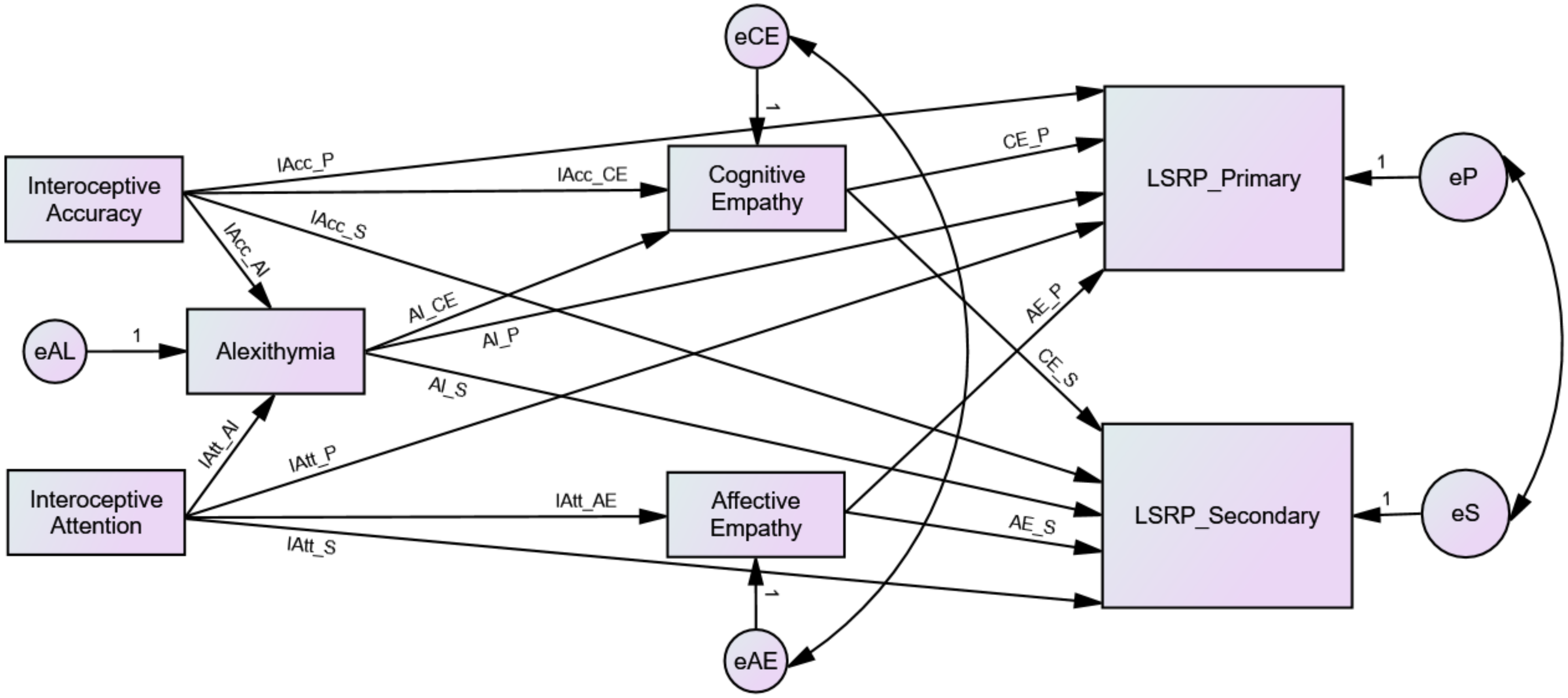

Goodness of fit statistics

$\chi^2(4) = 26.542$; $p < .001$; RMSEA = .105; RMSEA 90% CI = [.069, .144]; TLI = .743; CFI = .951; AIC = 74.542; BIC = 176.402

Table 5. *Direct effects, correlations and squared multiple correlations for Model 1B*

**Standardized Direct Effects (Regression Weights)**			**Estimate**	**99% CI Lower Limit**	**99% CI Upper Limit**	***p***
TAS_TotalScore	<---	BPQ_BodyAwareness	.031	-.074	.133	.456
TAS_TotalScore	<---	IAS_TotalScore	-.297	-.403	-.183	<.001
QCAE_CognitiveEmpathy	<---	IAS_TotalScore	.260	.156	.361	<.001
QCAE_CognitiveEmpathy	<---	TAS_TotalScore	-.269	-.369	-.160	<.001
QCAE_AffectiveEmpathy	<---	BPQ_BodyAwareness	.096	.000	.195	.010
LSRP_Primary	<---	BPQ_BodyAwareness	-.030	-.140	.084	.502
LSRP_Secondary	<---	TAS_TotalScore	.402	.295	.500	<.001
LSRP_Primary	<---	QCAE_CognitiveEmpathy	-.125	-.253	.004	.013
LSRP_Primary	<---	IAS_TotalScore	.019	-.103	.145	.702
LSRP_Secondary	<---	IAS_TotalScore	.061	-.059	.176	.186
LSRP_Secondary	<---	BPQ_BodyAwareness	.041	-.052	.139	.250
LSRP_Primary	<---	TAS_TotalScore	.128	.014	.241	.005
LSRP_Secondary	<---	QCAE_CognitiveEmpathy	-.209	-.331	-.079	<.001
LSRP_Primary	<---	QCAE_AffectiveEmpathy	-.255	-.360	-.145	<.001
LSRP_Secondary	<---	QCAE_AffectiveEmpathy	.111	.007	.219	.006
**Correlations**			**Estimate**	**99% CI Lower Limit**	**99% CI Upper Limit**	***p***
eCE	<-->	eAE	.338	.230	.440	<.001
eS	<-->	eP	.266	.151	.386	<.001
**Squared Multiple Correlations**			**Estimate**	**99% CI Lower Limit**	**99% CI Upper Limit**	***p***
		TAS_TotalScore	.089	.034	.161	<.001
		QCAE_AffectiveEmpathy	.009	.000	.038	<.001
		QCAE_CognitiveEmpathy	.181	.104	.268	<.001
		LSRP_Primary	.126	.053	.199	.002
		LSRP_Secondary	.246	.150	.330	.002

Table 6. *Specific Indirect Effects for Model 1B*

Specific Indirect Effects (Unstandardized Weights)	Estimate	99% CI Lower Limit	99% CI Upper Limit	*p*
Interoceptive Accuracy ---> Primary Psychopathy Via Cognitive Empathy	-.018	-.041	.000	.009
Interoceptive Accuracy ---> Secondary Psychopathy Via Cognitive Empathy	-.022	-.041	-.009	<.001
Interoceptive Accuracy ---> Primary Psychopathy Via Alexithymia	-.021	-.045	-.003	.003
Interoceptive Accuracy ---> Secondary Psychopathy Via Alexithymia	-.048	-.075	-.027	<.001
Interoceptive Accuracy ---> Primary Psychopathy Via Alexithymia & Cognitive Empathy	-.005	-.013	.000	.007
Interoceptive Accuracy ---> Secondary Psychopathy Via Alexithymia & Cognitive Empathy	-.007	-.014	-.002	<.001
Interoceptive Attention ---> Primary Psychopathy Via Affective Empathy	-.006	-.016	.000	.008
Interoceptive Attention ---> Secondary Psychopathy Via Affective Empathy	.002	.000	.007	.008
Interoceptive Attention ---> Primary Psychopathy Via Alexithymia	.001	-.003	.006	.344
Interoceptive Attention ---> Secondary Psychopathy Via Alexithymia	.002	-.006	.011	.448
Interoceptive Attention ---> Primary Psychopathy Via Alexithymia & Cognitive Empathy	.000	-.001	.002	.325
Interoceptive Attention ---> Secondary Psychopathy Via Alexithymia & Cognitive Empathy	.000	-.001	.002	.382
Alexithymia ---> Primary Psychopathy Via Cognitive Empathy	.018	.000	.038	.009
Alexithymia ---> Secondary Psychopathy Via Cognitive Empathy	.022	.009	.041	<.001
Interoceptive Accuracy ---> Cognitive Empathy Via Alexithymia	.054	.027	.090	<.001

Modification indices: Covariances (IAS and BPQ Body Awareness)

The covariance between IAS and BPQ Body Awareness was included in model 2B.

Model 2B. Hypothesis-Driven Model Plus Selected Modification Indices for Classical Factors

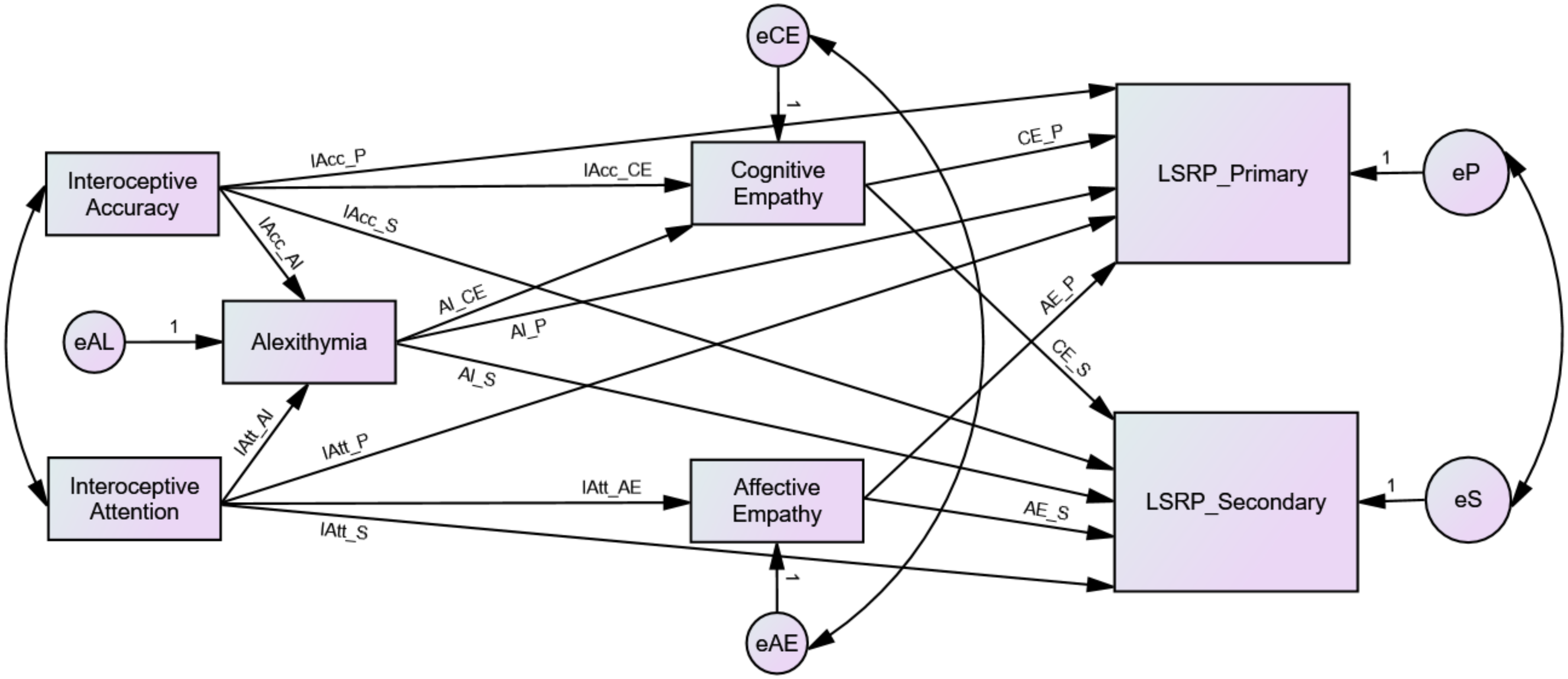

Goodness of fit statistics

$\chi^2(3) = 4.670$; $p = .198$; RMSEA = .033; RMSEA 90% CI = [.000, .087]; TLI = .975; CFI = .996; AIC = 54.670; BIC = 160.774

Table 7. *Direct effects, correlations and squared multiple correlations for Model 2B*

**Standardized Direct Effects (Regression Weights)**			**Estimate**	**99% CI Lower Limit**	**99% CI Upper Limit**	***p***
TAS_TotalScore	<---	BPQ_BodyAwareness	.031	-.074	.134	.456
TAS_TotalScore	<---	IAS_TotalScore	-.297	-.404	-.183	<.001
QCAE_CognitiveEmpathy	<---	IAS_TotalScore	.260	.156	.361	<.001
QCAE_CognitiveEmpathy	<---	TAS_TotalScore	-.269	-.368	-.159	<.001
QCAE_AffectiveEmpathy	<---	BPQ_BodyAwareness	.096	.000	.195	.010
LSRP_Primary	<---	BPQ_BodyAwareness	-.030	-.140	.084	.502
LSRP_Secondary	<---	TAS_TotalScore	.402	.296	.500	<.001
LSRP_Primary	<---	QCAE_CognitiveEmpathy	-.125	-.253	.004	.013
LSRP_Primary	<---	IAS_TotalScore	.019	-.103	.145	.702
LSRP_Secondary	<---	IAS_TotalScore	.061	-.059	.176	.186
LSRP_Secondary	<---	BPQ_BodyAwareness	.041	-.052	.139	.250
LSRP_Primary	<---	TAS_TotalScore	.128	.014	.240	.005
LSRP_Secondary	<---	QCAE_CognitiveEmpathy	-.209	-.331	-.079	<.001
LSRP_Primary	<---	QCAE_AffectiveEmpathy	-.255	-.360	-.145	<.001
LSRP_Secondary	<---	QCAE_AffectiveEmpathy	.111	.007	.219	.006

**Correlations**			**Estimate**	**99% CI Lower Limit**	**99% CI Upper Limit**	***p***
IAS_TotalScore	<-->	BPQ_BodyAwareness	.204	.082	.307	<.001
eCE	<-->	eAE	.338	.230	.440	<.001
eS	<-->	eP	.266	.151	.386	<.001

**Squared Multiple Correlations**	**Estimate**	**99% CI Lower Limit**	**99% CI Upper Limit**	***p***
TAS_TotalScore	.086	.031	.155	<.001
QCAE_AffectiveEmpathy	.009	.000	.038	<.001
QCAE_CognitiveEmpathy	.180	.102	.265	<.001
LSRP_Primary	.128	.054	.201	.002
LSRP_Secondary	.243	.149	.326	.002

Table 8. *Specific Indirect Effects for Model 2B*

**Specific Indirect Effects (Unstandardized Weights)**	**Estimate**	**99% CI Lower Limit**	**99% CI Upper Limit**	***p***
Interoceptive Accuracy ---> Primary Psychopathy Via Cognitive Empathy	-.018	-.041	.000	.009
Interoceptive Accuracy ---> Secondary Psychopathy Via Cognitive Empathy	-.022	-.041	-.009	<.001
Interoceptive Accuracy ---> Primary Psychopathy Via Alexithymia	-.021	-.045	-.003	.003
Interoceptive Accuracy ---> Secondary Psychopathy Via Alexithymia	-.048	-.075	-.027	<.001
Interoceptive Accuracy ---> Primary Psychopathy Via Alexithymia & Cognitive Empathy	-.005	-.013	.000	.007
Interoceptive Accuracy ---> Secondary Psychopathy Via Alexithymia & Cognitive Empathy	-.007	-.014	-.002	<.001
Interoceptive Attention ---> Primary Psychopathy Via Affective Empathy	-.006	-.016	.000	.008
Interoceptive Attention ---> Secondary Psychopathy Via Affective Empathy	.002	.000	.007	.008
Interoceptive Attention ---> Primary Psychopathy Via Alexithymia	.001	-.003	.006	.344
Interoceptive Attention ---> Secondary Psychopathy Via Alexithymia	.002	-.006	.011	.448
Interoceptive Attention ---> Primary Psychopathy Via Alexithymia & Cognitive Empathy	.000	-.001	.002	.325
Interoceptive Attention ---> Secondary Psychopathy Via Alexithymia & Cognitive Empathy	.000	-.001	.002	.382
Alexithymia ---> Primary Psychopathy Via Cognitive Empathy	.018	.000	.038	.009
Alexithymia ---> Secondary Psychopathy Via Cognitive Empathy	.022	.009	.041	<.001
Interoceptive Accuracy ---> Cognitive Empathy Via Alexithymia	.054	.027	.090	<.001

Modification indices: None
